# Metagenomics of Toenail Onychomycosis in Three Victorian Regions of Australia

**DOI:** 10.3390/jof8111198

**Published:** 2022-11-14

**Authors:** Steven Hainsworth, Ann C. Lawrie, Thiru Vanniasinkam, Danilla Grando

**Affiliations:** 1 School of Science, RMIT University (Bundoora Campus), P.O. Box 71, Bundoora, VIC 3083, Australia; 2 School of Biomedical Sciences, Charles Sturt University, Wagga Wagga, NSW 2650, Australia

**Keywords:** metagenomics, ITS2, 16S onychomycosis, tinea unguium, dermatophyte, *Trichophyton*, *rubrum*, *mentagrophytes*, *T. interdigitale/mentagrophytes*, *interdigitale*, Next generation sequencing

## Abstract

Onychomycosis is a fungal disease of the nail that is found worldwide and is difficult to diagnose accurately. This study used metagenomics to investigate the microbiology of 18 clinically diagnosed mycotic nails and two normal nails for fungi and bacteria using the ITS2 and 16S loci. Four mycotic nails were from Bass Coast, six from Melbourne Metropolitan and eight from Shepparton, Victoria, Australia. The mycotic nails were photographed and metagenomically analysed. The ITS2 sequences for *T. rubrum* and *T. interdigitale/mentagrophytes* averaged over 90% of hits in 14/18 nails. The high abundance of sequences of a single dermatophyte, compared to all other fungi in a single nail, made it the most likely infecting agents (MLIA). *Trichophyton rubrum* and *T.* interdigitale/mentagrophytes were found in Bass Coast and Shepparton while only *T. interdigitale/mentagrophytes* was found in Melbourne. Two nails with *T. interdigitale/mentagrophytes* mixed with high abundance non-dermatophyte moulds (NDMs) (*Aspergillus versicolor*, *Acremonium sclerotigenum*) were also observed. The two control nails contained chiefly *Fusarium oxysporum* and *Malassezia slooffiae*. For bacteria, *Staphylococcus epidermidis* was in every nail and was the most abundant, including the control nails, with an overall mean rate of 66.01%. *Rothia koreensis*, *Corynebacterium tuberculostearicum*, and *Brevibacterium sediminis* also featured.

## 1. Introduction

Onychomycosis is a global disease phenomenon occuring in approximately 2–10% of the general population [1]. The incidence increases with age to reach 50% at over 70 years old [2] and is more prevalent in at risk groups such as diabetics [3]. The direct cost of medications for dermatophytes alone was estimated globally at US$5 billion in 1997 [4] and at US$ 1671 m in 2004 in USA alone [5]. The term ‘onychomycosis’ is used for a fungal infection of the nail including non-dermatophyte moulds (NDMs) or yeasts, or if the infecting organism is unknown. If a diagnosis of a dermatophyte infection occurs the condition is then considered to be tinea unguium in line with the nomenclature of tinea pedis, tinea corporis etc.. Three groups of fungi may be detected individually or in combination: dermatophytes, NDMs and yeasts [6,7]. About 80–90% of toenail cases of mycotic infection are caused by dermatophytes [8,9,10] and the remainder by NDMs and yeasts [11]. Dermatophytoses are infections of the skin, hair and nails caused by dermatophytes and affect 20–25% of the world’s population [12]. They are the most common fungal infections in the world and are increasing in frequency [13]. The two most common dermatophytes in humans are *T. rubrum* and *T. interdigitale* [14,15,16,17]. The ITS2 region used in this study is unable to differentiate between the two species *T. interdigitale and T. mentagrophytes* and was identified as *T. mentagrophytes* in the bioinformatics. For this reason the fungus is designated *T. interdigitale*/*mentagrophytes* in the text.

Worldwide, the greatest incidence of infection by dermatophytes occurs in the foot [18], in three sites: the plantar surface and interdigital spaces (tinea pedis) and the nails (tinea unguium), the last of which is the most common and most persistent [19,20,21,22]. Toenail onychomycosis is a fungal infection of any or all parts of the toenail unit, including the nail bed [23]. Approximately 80% of infections are in the great toenail and globally, 60–90% of all toenail onychomycoses are caused by dermatophytes [8,10,11,16,24,25]. The role of non-dermatophyte fungi has been controversial, with the role of bacteria unknown. The isolation of NDMs but no dermatophytes from diseased nails is frequently considered to be contamination [26,27] but other authors rank several NDMs such as *Scopulariopsis*, *Fusarium* and *Aspergillus* species as pathogens [28,29].

Until recently the gold standard for mycotic nail diagnosis was the culturing of a dermatophyte which was then identified by traditional morphological and physiological methods [27,30,31]. Subsequently identification by PCR methods [32] including ITS sequencing have been recognised as being more accurate [33,34]. The growth of a culture was considered as *prima facie* evidence that a dermatophyte was the causative organism. However, the low culturing success rate of dermatophytes from nail specimens is well documented and consequently many tinea unguium nails processed in the laboratory lack certainty of diagnosis when no dermatophyte is cultured [35].

To overcome the limitations associated with culture, various PCR methods have been proposed to work directly from the nail tissue [35] but the majority of these give no data on the abundance of the identified dermatophyte. However, the most promising direct PCR approach for dermatophytes is by Iwanaga et al. (2017, 2020), where the abundance of dermatophyte ITS sequences can be estimated before and during treatment [36,37]. While these specific primers can locate a variety of microorganisms, they cannot fully explore the microbiology of the nail, for which metagenomics is required.

Metagenomics uses Next Generation Sequencing (NGS) and bioinformatics to study the genetic material of microorganisms directly (without culture), from environmental and biological samples. The DNA within the sample is extracted, purified and quantified. Primers for the selected loci are used to sequence target areas such as the ITS2 and 16S regions, which results in amplicons of up to 300 bp long from the DNA locus of the primers. These overlapping reads are assembled to produce de novo sequences from the original DNA. Bioinformatics is then used to identify microorganisms from these sequence assemblies by comparison with public databases. To increase accuracy, this should include TYPE and reference strains of the identified species. Metagenomics gives insight into the relative abundance of individual species within the global community of organisms present in the sample (Temperton 2012, Tomic-Canic 2014) and can be used to investigate any combination of fungi, bacteria, archaea and viruses. Thus, even small proportions of a microorganism in a specimen can be found and identified. Metagenomics has been used extensively to identify microorganisms in their communities and in humans it has been applied to the gut [38,39,40] and skin biomes [18,41].

To date only two metagenomic studies have been published on onychomycosis [16,42]. Cruz-Correa et al. (2016), studied only one nail with a known *T. rubrum* infection for fungi and bacteria. Joyce et al. (2019), although using a large sample of 8816 clinically suspicious nails in North America, analysed at a metadata level with no consideration of individual nail infections or their clinical presentations. To date, the findings have confirmed the traditional understanding that a wide range of fungi (dermatophytes, NDMs and yeasts) and bacteria (predominantly *Staphylococcus* spp.) are present in mycotic nails [26,29,43]. Normal control nails had no fungi and but did have bacteria, especially *S. epidermidis* [16]. However, there have been no systematic studies of the metagenomics of individual diseased toenails or of normal toenails. Such a study could find if dermatophytes as well as NDMs and/or yeasts are present and so help to clarify the role of NDMs as primary or secondary pathogens following primary invasion by dermatophytes [29].

The aim of this study was to improve our understanding of onychomycosis by using metagenomics to give a microbial fingerprint of an individual nail for which patient details and morphological nail signs have been recorded photographically. This study is the first of its kind and addresses gaps in the knowledge of onychomycosis by using fungal and bacterial NGS analysis of 18 photographed mycotic nails and two normal control nails.

## 2. Materials and Methods

This study was conducted under approval number HREC 110–19/22622 at RMIT University. Informed consent was obtained from all subjects involved in the study. Twenty samples of nail clippings and scrapings were collected from three separate temperate areas of the State of Victoria: Bass Coast (coastal, 4 mycotic nails), Melbourne Metropolitan (large city, 6 mycotic nails plus 1 control) and Shepparton (rural town and surrounds, 8 mycotic nails plus 1 control) by practising podiatrists. Eighteen of the samples were clinically diagnosed as ‘onychomycosis’; two were judged not to be mycotic and suitable as controls. Except for one control nail, the nails were photographed, and the type of onychomycosis diagnosed according to the clinical appearance. The age of the patients was also recorded (Figure S1).

Each nail, prior to sampling, was swabbed with 0.5% chlorhexidine in 70% ethanol in sterile water. The nail samples of clippings and debris were collected into separate sterile 5 mL Eppendorf tubes by the participating podiatrists (Figure S2A) then transferred to 1.5 mL Eppendorf tubes for DNA extraction (Figure S2B). DNA was extracted using an Epicentre MasterPure Yeast DNA Purification Kit (Astral, Taren Point, Australia) according to the manufacturer’s protocol. This was quantified using a spectrophotometer (LVis, BMG LABTECH GmbH Polarstar Omega 0415, Germany) according to the manufacturer’s instructions. The DNA was sent to Macrogen Oceania (https://www.macrogen.com.au/ URL accessed on 15 December 2020) and metagenomic analysis was performed by Macrogen in Seoul, South Korea. Prior to analysis of the entire batch, Macrogen performed DNA quality control using the Picogreen method and Victor 3 fluorometry and the condition of the DNA was assessed by gel electrophoresis. DNA was amplified using MiSeq 300bp PE for Next Generation Sequencing (NGS) for the target loci (the ITS2 region for fungi and the 16S region for bacteria) and produced an amplicon DNA library for each sample. The expected output range was 50–100 K reads per amplicon of raw data. Primers used were ITS3 + ITS4 (White et al., 1990) to target the ITS2 rRNA region for fungi and Bakt_341 + Bakt_805 (Klindworth 2013) to target the 16S rRNA region for bacteria (Table S1).

For the ITS2 locus, raw sequence data, Operational Taxonomic Units (OTUs), bioinformatics and UNITE database (UNITE (ut.ee)) identifications were downloaded from the Macrogen server. For the 16S locus, raw sequence data, OTUs and bioinformatics for bacteria were also downloaded from the server, but identifications were obtained from GenBank through NCBI (National Center for Biotechnology Information (nih.gov)). The default database used by Macrogen for the ITS2 region was UNITE, which contains not only sequences for medical fungal species but for many other eukaryotic organisms. The UNITE database did not identify the dominant fungus in Nails 10 and 11 but it was identified in GenBank as *Fusarium oxysporum*.

**Figure 1:**
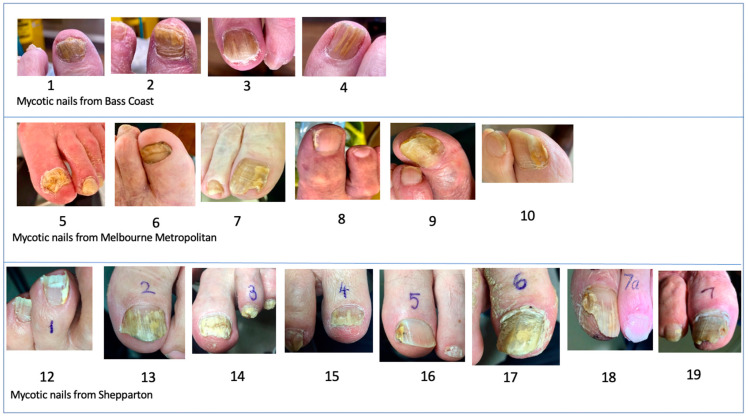
Images of 18 toenails clinically diagnosed as mycotic. Numbers drawn on the toes were solely for the purpose of identification by the contributing podiatrist. Missing numbers 11 and 20 were normal control nails.

The results of each locus with the corresponding samples were arranged in tables in descending order of abundance (Tables S2 and S3). Organisms that had less than 0.01% DNA were not processed but listed as “Miscellaneous Unidentified”. From these tables stacked histograms of the top ten organisms for the ITS2 region and the top five for the 16S region were created. The results of the ITS2 histograms indicated the MLIA as shown in Figure 1.

Principal Components Analysis (PCA) was used to analyse ITS2 results for fungi and group nails dominated by *T. rubrum*, *T. interdigitale/mentagrophytes* and/or an NDM. It was also used similarly for the 16S sequences. Statistical analyses were conducted in the statistical program Minitab^®^ version 21.1.1 (www.minitab.com).

## 3. Results

### 3.1. Fungi

All ITS2 counts were above 100 K, exceeding the required Illumina Miseq read counts of 50–100 K (Figure S3A). The read quality was high with Q-Phred scores at Q-20 and Q-30 (Figure S3A,B). The complete taxonomic results for ITS2 are in Table S2.

**Figure 2:**
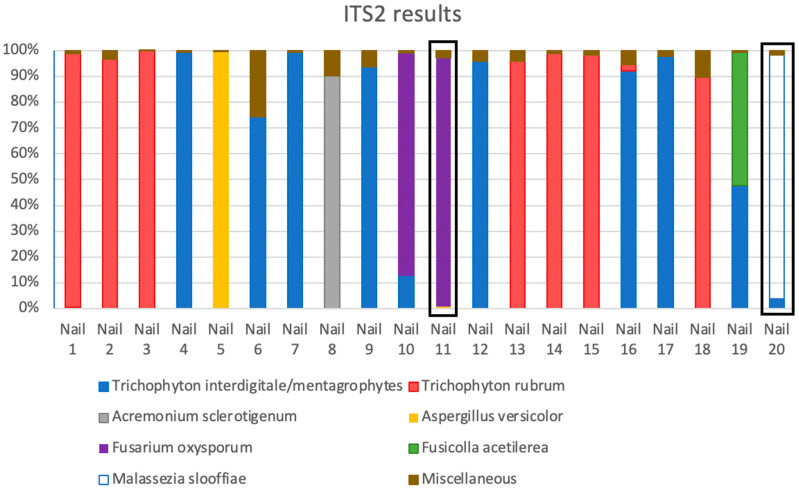
Main fungal species detected in mycotic and control nails. Stacked histograms show dominant species results of ITS2 for all nails. Normal control nails are boxed in black.

All nails showed high concentrations of at least one fungus (Figure 2, Table S2). The DNA of a large array of NDMs and yeasts was found in all the nails. Most of these were in trace concentrations (below 1%). One fungus predominated over all other fungi in each nail except in Nails 10 and 19. These nails (10 and 19) both contained significant amounts of *T. interdigitale/mentagrophytes* (12.81% and 47.55% respectively) along with noteworthy quantities of only one NDM either *Fusarium oxysporum* (86.27%) or *Fusicolla acetilerea* (51.92%) respectively (Figure 2). Seven nails were dominated by *T. interdigitale/mentagrophytes* (Nails 4,6,7,9,12,16,17), seven by *T. rubrum* (Nails 1,2,3,13,14,15,18) and two contained *T. interdigitale/mentagrophytes* (Nails 10, 19) mixed with an NDM: Nail 10 had 13% *T. interdigitale/mentagrophytes* with 86% *Fusarium oxysporum* whereas Nail 19 had 48% *T. interdigitale*/*mentagrophytes* with 52% of *Fusicolla acetilerea*. The dominant NDMs for the mycotic nails were *Aspergillus versicolor* (Nail 5), *Acremonium sclerotigenum* (Nail 8) and *F. oxysporum* (Nail 10). For the ‘normal’ control nails, the dominant fungi were the NDM *F. oxysporum* (Nail 11) and the yeast *Malassezia slooffiae* (Nail 20) (Figure 2).

The number of hits per nail ranged from 5–136 identifications with a mean of 39.4 hits per nail. These hits ranged from 0.01–99.94% of the total hits per nail. Only seven fungi had a mean per nail of over ≥1% of the total hits. These were two dermatophytes (*T. rubrum* and *T. interdigitale/mentagrophytes*), four NDMs (*Acremonium sclerotigenum, Aspergillus versicolor*, *Fusarium oxysporum* and *Fusicolla acetilerea*) and the yeast *Malassezia slooffiae* (Table S2).

There were three clusters of nails by principal components analysis (PCA), dominated respectively by *T. rubrum* and *T. interdigitale/mentagrophytes* and the NDMs (Figure S4). In each case there was significant correlation with the others dominated by the same fungus. The nails dominated by *T. rubrum* were from Bass Coast (Nails 1–3) and Shepparton (Nails 13–16 plus 18) whereas those dominated by *T. interdigitale/mentagrophytes* were from all three localities. Melbourne Metropolitan had no nails dominated by *T. rubrum.*

The clinical photographs of the nails were grouped according to the highest scoring fungus found in each specimen (Figure S5). In the diseased nails there were eight dominated by *T. interdigitale/mentagrophytes*, seven dominated by *T. rubrum* and three nails dominated by NDMs (*Aspergillus versicolor*, *Acremonium sclerotigenum* or *F. oxysporum*). The dominant fungi for the controls were *Fusarium oxysporum* and *Malassezia slooffiae*. For the mycotic nails there was no visible perceptible difference in the signs or severity according to the dominant fungus, as all groups had crumbling and yellow discolouration, dermatophytomas and varying degrees of skin involvement.

Only two dermatophytes were identified in this metagenomic study, namely *T. interdigitale*/*mentagrophytes* and *T. rubrum.* Two NDMs found in high concentrations in diseased nails were *Asp. versicolor* (99.40%) and *Acr. sclerotigenum* (86.27%) in Nails 5 and 8 respectively. *Fusarium oxysporum* in Nail 10 was also in a high concentration of 86.27% but in co-existence with *T. interdigitale*/*mentagrophytes* at 12.81% of all hits. Nail 11 (Control 1) also contained a high concentration of *F. oxysporum* (95.77%). Nail 20 (Control 2) had *Malassezia slooffiae* as the dominant species (93.57%).

Five of the 14 nails dominated by either *T. rubrum* or *T. interdigitale*/*mentagrophytes* in concentrations higher than 98%, also contained the alternate species in trace quantities (0.01–0.65%) (Table 1). The mycotic Nail 10 which was dominated by *F. oxysporum* had both dermatophytes with *T. interdigitale*/*mentagrophytes* at 12.81% and *T. rubrum* in a trace amount. In addition to this, the control Nail 11 which was also dominated by *F. oxysporum*, contained small traces of both *T. rubrum* and *T. interdigitale*/*mentagrophytes*. Hence five (27.78%) of the 18 mycotic nails and 50% of the controls contained traces of both dermatophyte species (Tables S4 and S5). Further to this control Nail 20 which was dominated by DNA from *M. slooffiae* also contained 3.89% of *T. interdigitale*/*mentagrophytes* but no *T. rubrum* (Table 1).

The specimens from the Bass Coast region had both the dermatophytes but no NDMs as the MLIA in a ratio of 75% *T. rubrum*. Melbourne Metropolitan had *T. interdigitale*/*mentagrophytes* as the only dermatophyte but three NDMs as the MLIA, while Shepparton had an even mixture of the two dermatophytes as the MLIA with one unusual NDM Fusicolla acetilerea (syn. *Fusarium merismoides* var. *acetilereum*).

### 3.2. NDMs and Yeasts

The DNA of a large array of NDMs and yeasts was found in all the nails. Most of these were in small or trace concentrations below 1%. In the clinically diagnosed mycotic nails *Asp. versicolor*, *Acr. sclerotigenum* and *F. oxysporum* contained concentrations > 85%. In the control nails *F. oxysporum* and *M. slooffiae* were high scoring being >90%. (Table S2).

When considering the MLIA, the Bass Coast, a seaside regional area, had three cases of *T. rubrum* and one case of *T. interdigitale*/*mentagrophytes*; Melbourne Metropolitan a city area, had four *T. interdigitale*/*mentagrophytes*, two NDMs and one mixed *T. interdigitale*/*mentagrophytes* with an NDM; Shepparton, an inland country region had four *T. rubrum*, three *T. interdigitale*/*mentagrophytes* and one mixed *T. interdigitale*/*mentagrophytes* with an NDM (Figure 2; Table S2).

#### 3.2.1. Bacteria

All 16S Read counts were above 100 K, exceeding the required Illumina Miseq read counts of 50–100 K (Figure S6A). The Read quality was high with Q-Phred scores at Q-20 and Q-30 (Figure S6B). The complete taxonomic results for 16S are found in Table S3.

**Figure 3:**
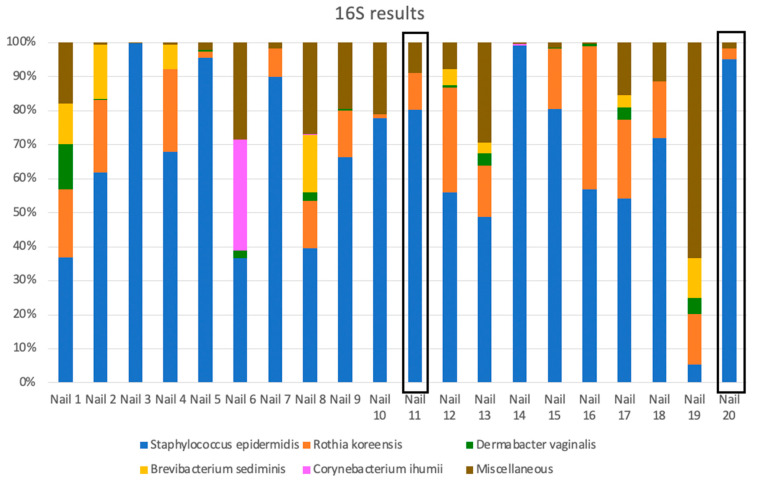
Main bacteria found in mycotic and normal control nails. Stacked histograms show dominant species results of 16S region analysis for all nails. Normal control nails are boxed in black.

The top five results for the bacteria comprised well over 80% of the total in most nails (Figure 3, Table S3). *Staphylococcus epidermidis* DNA was found in every nail, including the two controls and constituted an average of 67% of sequence assemblies in all nails, including the controls. Other common bacteria were *Rothia koreensis* (average of 14%), *Brevibacterium sediminis* (4%), *Corynebacterium tuberculostearicum* (3%), *C. ihumii*, *Dermabacter vaginalis* and *Moraxella osloensis* (all 2%) and *C. jeikeium*, *C. massiliense and C. resistens* (all 1%).

Results from 16S amplification showed a total of 125 identifications (species or other taxonomic classifications) over the 20 nails. The number of hits per nail ranged from 5–43 (0.01–66.02%) identifications per nail with a mean of 23.45 (Table S3). The percentage of hits in each nail ranged from 0.01–61.09% with an average of 1.10%. Only eleven bacteria had an average per nail of ≥1% of the total hits. The main bacterium was *S. epidermidis* in all nails, constituting two-thirds of the sequences found except for Nail 19 (34% *Moraxella osloensis*).

There were five clusters of nails by PCA, dominated respectively by each of the subdominant genera, including two species of *Corynebacterium* (Figure S7). In each case there was significant correlation with others dominated with the same subdominant bacterium (Table S6).

#### 3.2.2. Combined Overview of Microflora

**Figure 4:**
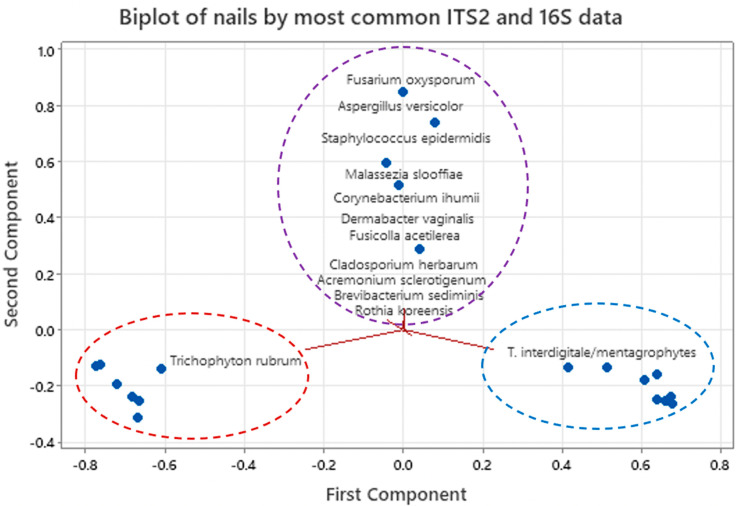
Biplot from Principal Components analysis of combined ITS2 and 16S data.

The relative proportions of hits for both ITS2 and16S declined sharply after the top few sequence assemblies for both, and subsequent hits ranged down rapidly to <1% of the totals. The data from the top eight fungi and top five bacteria were combined and analysed by PCA with covariance. This showed that nails separated into three main groups (Figure 4). One cluster formed around *T. rubrum*, a second around *T. interdigitale*/*mentagrophytes* and a third around nails with other fungi and all bacteria. This showed that the two dermatophytes were the greatest influence on the microflora and the NDMs, yeasts and bacteria less so.

## 4. Discussion

This study is the first to use metagenomics to explore fully the fungal and bacterial microflora of a series of individual nails. As such it has increased our understanding of the richness of the microcosm in individual nails, which is vital for appropriate clinical treatment and helps to determine the relative roles of each microorganism. The mycotic nails were typically dominated by one species of fungus (dermatophyte or NDM) that produced over 90% of the hits and the range of similar appearances demonstrated that clinical appearance was not a reliable guide as to the dominant fungus. Various skin-associated bacteria (principally *S. epidermis*) were also prevalent in the nails and may play a part in the disease process. Importantly, this study has shown that more than one potentially pathogenic fungus was present even in asymptomatic ‘normal’ nails, suggesting that the disease state may not always be inevitable. The sharing of individual nails by relatively large proportions of a dermatophyte and an NDM, as in nails 10 and 19, may indicate that the dermatophyte is the primary pathogen but is overwhelmed by the NDM.

### 4.1. Most Likely Infecting Agents (MLIAs)

When an anthropophilic dermatophyte such as *T. rubrum* or *T. interdigitale*/*mentagrophytes* is cultured from a mycotic nail sample it is assumed to be the infecting agent [35]. Similarly, when an anthropophilic dermatophyte is identified in high abundance using metagenomics, it is reasonable to assume that it is the most likely infecting agent (MLIA).

An important finding from this research is that it is highly likely that one fungus predominates when there is a clinical infection. In this study, most dermatophytes were detected at greater than 80% of sequence assemblies in individual nails and so are assumed to be the most likely infecting agent (MLIA). Of the 18 mycotic nails, 16 (89%) were dominated by dermatophytes, which is in accordance with the current global estimates of 90% [10,44]. Only two nail infections (11%) were possibly caused by NDMs and none by yeasts [29]. This study confirmed that clinical appearance cannot be used to identify the infecting agent at a macroscopic level [45,46].

The results of this metagenomic study demonstrates that *T. rubrum* and *T. interdigitale*/*mentagrophytes* were found exclusively as the MLIA in tinea unguium in these regions. Where one of these dermatophytes predominated in a diseased nail, the alternate one was found in smaller concentrations in 33.33% of the nails, albeit often in trace amounts. This may go some way to understanding the recalcitrant nature of tinea unguium, as the eradication of one species may lead to infection by the other species, especially if antifungal resistance is present [47]. Strain switching within the species *T. rubrum* has been observed with terbinafine treatment and also, to a lesser extent, in a placebo group [48]. It is also possible that species switching may occur between species in nails.

### 4.2. Mixed Infections

A question arises about NDMs found in very high abundance in nails without any detected dermatophyte, such as Nail 5 (99.4% *Asp. versicolor*) and Nail 8 (86% *Acr. sclerotigenum*). Both species have previously been recorded as nail pathogens. *Asp. versicolor* was documented in nails by some investigators [49,50] and as being able to degrade some parts of the nail in vitro [51,52]. *Acr. sclerotigenum* has also previously been recorded as a nail pathogen [53,54,55]. The high abundance of both fungi makes them the most likely candidates to be the infecting agents. However, it is possible that initially a dermatophyte infected the nail but was overtaken by these fungi. The presence of large quantities of both *T. interdigitale*/*mentagrophytes* and an NDM (*Fusicolla acetilerea* in Nail 10 and *Fusarium oxysporum* in Nail 20) strongly suggests double occupancy of single nails. Both these fungi belong to the *Fusarium oxysporum* complex [56].

Nail 10 may be a mixed infection between *F. oxysporum* and *T. interdigitale*/*mentagrophytes*, although it could be argued that *F. oxysporum* played very little role as it was also predominant as natural flora (96%) in the non-diseased control Nail 11. It is also possible that Nail 10 is a *T. interdigitale*/*mentagrophytes* infection that has been colonised by *F. oxysporum* (or vice versa). Several researchers assert that *F. oxysporum* is a pathogen in onychomycosis [57,58]. Veiga et al., 2018 demonstrated using an ex vivo model of human nail fragments that *F. oxysporum* invaded the nail and formed a biofilm. They concluded that *F. oxysporum* was therefore a likely primary pathogen [59]. However, the results of this metagenomic study do not substantiate these assertions. In Nail 11, a control nail, there were no clinical signs of infection despite 96% occupancy by *F. oxysporum*, indicating that the fungus could be a commensal but changes in its role over time or in the presence of a dermatophyte.

The finding of this study for the control nails 11 and 20, was that there was an abundance of NDMs and yeasts with most being less than 1%. Nail 11 also contained *T. interdigitale*/*mentagrophytes* and *T. rubrum* in trace amounts while Nail 20 had *T. interdigitale*/*mentagrophytes* as 3.89% of the total hits. Each of these nails were dominated by an NDM (Nail 11) or a yeast (Nail 20). By contrast, Joyce et al. (2019) found using metagenomics that 20 twenty normal control nails had no fungi [16] (although those results could have been affected by the sensitivity and cut-offs of the method used). Nowicki et al. (2016) cultured fungi from the dust of 36 of 77 (46.8%) normal-appearing nails. Eight of these were dermatophytes (six *T. rubrum* and two *T. interdigitale*/*mentagrophytes*) [60]. The concentration of these dermatophytes in the nail dust is unknown.

### 4.3. Significance of NDMs, Yeasts and Bacteria in Nails

A large range of NDMs has previously been found within infected nails by culture [26] and metagenomics [16,42]. Ellis et al. (1997) listed 22 NDMs and two yeasts grown in culture from 118 nails infected with dermatophytes, of which this metagenomic study found 63% in common. Both studies support the idea that there are numerous NDMs, and yeasts present in mycotic nails. The three most common NDM genera identified by Ellis et al. (1997) were *Cladosporium*, *Alternaria* and *Epicoccum* [26], all of which were found in these nails studied by metagenomics.

The role of non-dermatophytes in tinea unguium is little understood [46]. Some may be primary pathogens [61] or secondary invaders and contaminants [29]. Although speculative, some species under the right conditions may become primary pathogens, as in Nails 5 and 8 [29]. Ellis et al. (1997) found that the presence of NDMs did not affect the outcome when terbinafine treatment is applied [26]. This may be because they are contaminants, play a minor role or possibly because many of them are also susceptible to TRB [62]. Further research is needed to clarify the aetiology of these fungi in nails. With one exception, yeasts other than *Candida* were in trace amounts. The exception was Nail 20, which was a control ‘normal’ nail and had 94% of the skin commensal *M. slooffiae*, by contrast with other nails that had less than 1%. The yeast *Rhodotorula* sp. occurred in Nail 6 at 1.21% but below 1% in other nails. These yeasts are unlikely to play any role as toenail pathogens in the nails in this study, except perhaps as saprophytes [26].

There was a large array of bacterial DNA found in the nails, but most species found in the mycotic nails were in trace amounts. The role, if any, of such a wide variety of bacterial genera and species is not understood. Some may be passively embedded in the nail. *Staphylococcus epidermidis* is the predominant bacterium and forms sticky biofilms that may assist in the adhesion of other microorganisms. However, it is possible, particularly in those with a high percentage such as *S. epidermidis*, that they obtain nutrients from the products produced by the fungi, especially the dermatophytes. Some species of *Staphylococcus* and *Brevibacterium*, including *S. epidermidis,* are able to degrade skin keratin [63]. Also *Corynebacterium* species break down, tunnel holes and fragment the keratin in the stratum corneum of the plantar skin of the foot, resulting in Pitted Keratolysis [64] and may be able to degrade toenail keratin in a similar fashion.

### 4.4. Limitations of This Study

Whilst this study reveals the most abundant organisms in the mycotic and control nails used, its usefulness is limited by the relatively small numbers of nails analysed, especially only two ‘normal’ nails without visible symptoms. It is also limited by only using metagenomics with ITS2 because it does not distinguish between *T. interdigitale* and *T. mentagrophytes*, for which metagenomics with ITS1 would be required, at extra cost. Other limitations include the relatively restricted geographical area (Victoria, Australia) and population studied (patients attending podiatrists). More detailed analysis of individual nails in a larger population would allow the conclusions inferred here to be tested more critically.

### 4.5. Estimated Costs of Metagenomic Analysis

For further researchers the following is an estimated cost per locus from the provider Macrogen in October 2022. In Australian dollars: ITS sequencing, generating ~100 K read per sample = AUD120 per sample. Bioinformatics for between 10 to 20 samples is ~AUD50-100 per sample. The costs differ depending on the number of samples being sent to the provider, so a quotation should be sought. The estimated turnaround time is 2 to 4 weeks.

## 5. Conclusions

Metagenomics using the ITS2 and 16S regions was highly sensitive for fungal and bacterial identification and quantification. The ITS2 and 16S regions proved powerful in finding and identifying sequence assemblies of 354 fungi and 125 bacteria found in 18 mycotic nails and two normal nails. Most nails were over 90% occupied by one fungus. The main pathogens were the dermatophytes *T. interdigitale*/*mentagrophytes* and *T. rubrum* but over 90% of two nails was occupied by NDMs. Mixed occupancy by dermatophytes, NDMs, yeasts and bacteria were common. Most NDMs and yeasts were found in only small quantities in mycotic and normal nails. *Staphylococcus epidermidis* was the most prominent bacteria and was found in all nails. Metagenomics proved useful in providing quantitative information on infections in individual nail, which could be useful in diagnosing and prescribing treatment for the condition as well as in epidemiological studies. Further research involving higher case numbers of onychomycosis should be conducted to confirm these findings.

## Figures and Tables

**Table 1:** Nails with *Trichophyton* species present as non-dominant fungus.

Nail Number	Dominant Fungus	Dermatophyte Trace
1	98.11% *T. rubrum*	0.65% *T. interdigitale*/*mentagrophytes*
3	99.93% *T. rubrum*	0.02% *T. interdigitale*/*mentagrophytes*
4	99.13% *T. interdigitale*/*mentagrophytes*	0.01% *T. rubrum*
7	99.45% *T. interdigitale*/*mentagrophytes*	0.02% *T. rubrum*
10	86.27% *F. oxysporum*	12.81% *T. interdigitale*/*mentagrophytes* 0.01% *T. rubrum*
11 (control)	95.77% *F. oxysporum*	0.08% *T. rubrum* 0.06% *T. interdigitale*/*mentagrophytes*
12	94.73% *T. interdigitale*/*mentagrophytes*	0.02% *T. rubrum*
20 (control)	93.57% *Malassezia slooffiae*	3.89% *T. interdigitale*/*mentagrophytes*

## Data Availability

The data presented in this study are available on request from the corresponding author. The data are not publicly available due to privacy concerns.

## Supplementary Materials

The following supporting information can be downloaded at: https://www.mdpi.com/article/10.3390/jof8111198/s1, Figure S1: Characteristics of samples used in the study. Nails were classified using the clinical appearance and classified as follows: DLSO: distal subfungal onychomycosis. SWO: superficial white onychomycosis. TDO: total dystrophic onychomycosis. *When the exact age was unknown the midpoint in the decade was used for calculations e.g. 80s becomes 85. Numbers drawn on the toes were solely for the purpose of identification by the contributing podiatrist; Figure S2: A Typical nail samples provided by podiatrists. Note the crumbling porous nature. B Samples after DNA extraction in 1.5 ml Eppendorf tubes. The two controls 11 (SJC) and 20 (SPC) show less pigmentation than infected nails.: 12 (SP1), 13 (SP2), 17 (SP6) and 19 (SP7); Figure S3: ITS2. A Read Counts B Phred scores. Sample read counts and sequence quality for ITS2 data. Read counts and Phred scores were all within the expected range (Reads 50–100K, Phred Q30 > 70–80%); Figure S4: PCA score plot of ITS2 results for dominant fungi; Figure S5: Clinical appearance of toenails grouped by MLIA. Trichophyton mentagrophytes (top), T. rubrum (middle) and an NDM (bottom) as the dominant fungus. Mycotic nails are 1–10 and 12–19; control nails are 11 and 20 (not pictured); Figure S6: Sample read counts (A) and sequence quality (B) from 16S primers. The top five results for the bacteria comprised well over 80% of the total population/ community detected in most nails. Staphylococcus epidermidis DNA was found in every nail including the two controls. Rothia koreensis was also prominent as was Brevibacterium sediminis (Table S5); Figure S7: Score plot from PCA, showing the separation of three clusters of nails characterised by the subdominant bacteria; Table S1: Primers used for sequencing by locus; Table S2: ITS2 rRNA Results; Table S3: 16S rRNA results; Table S4: Predominant organism percentage of hits, with most likely infecting agent (MLIA); Table S5: Nails with both T. rubrum and T. mentagrophytes present and showing the dominance of one dermatophyte over the other; Table S6: Correlations between results of sequencing the 16S region in DNA from nails.
