# Supplementary material for: Metagenomics of Toenail Onychomycosis in Three Victorian Regions of Australia
Source: J Fungi (Basel). 2022 Nov 14;8(11):1198. doi: 10.3390/jof8111198 (PMC9698484; doi:10.3390/jof8111198)
Supplement: Supplementary file 1 [file jof-08-01198-s001.zip › jof-1972601-Supplementary.pdf]

Supplementary Figures

Sample number	Clinical label	Geographic location	Clinical diagnosis /description	Age in years and Gender	Clinical Appearance
1	BC1	Bass Coast	Advanced DLSO. Dermatophytomas. Tinea pedis	77 Female	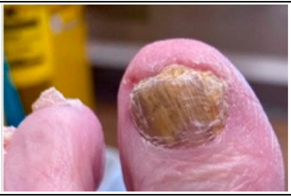
2	BC2	Bass Coast	DLSO. Dermatophytomas. Tinea pedis	87 Female	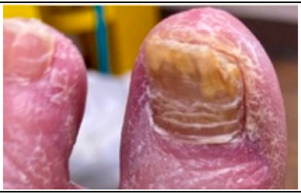
3	BC3	Bass Coast	DLSO. Dermatophytomas. Tinea pedis. Surrounding inflammation	71 Female	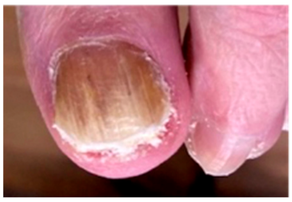
4	BC4	Bass Coast	DLSO. Dermatophytomas. Tinea pedis	77 Female	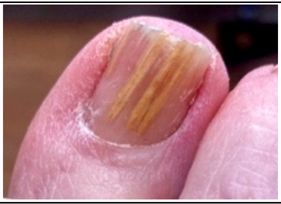
5	SJ1	Melbourne	TDO. Multiple nails. Tinea pedis	70s Female	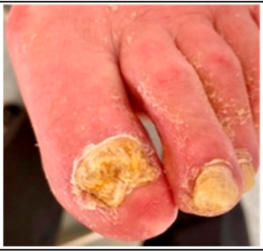
6	SJ2	Melbourne	DLSO. Limited mild tinea pedis	90 Female	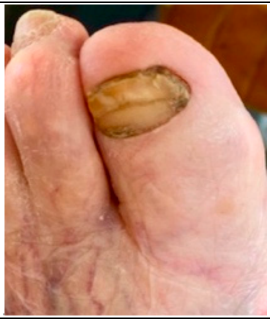

7	SJ3	Melbourne	DLSO. Distal inflammation	90s Female	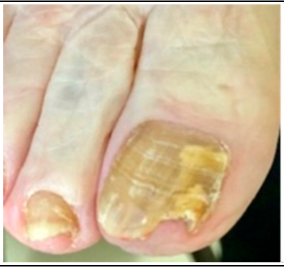
8	SJ4	Melbourne	Restricted DLSO with restricted tinea pedis	80s Male	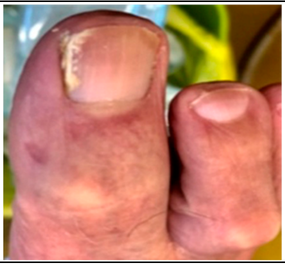
9	SJ5	Melbourne	Severe DLSO with intact ventral surface. Surrounding inflammation	90s Male	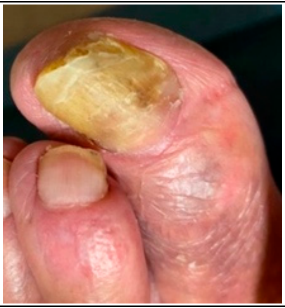
10	SJ6	Melbourne	DLSO with matrix involvement. Limited tinea pedis	80s Male	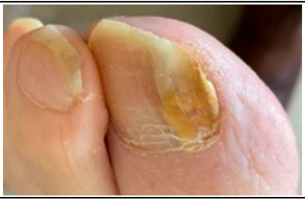
11	SJC (Control 1)	Melbourne	Not Available	Not Available	Not Available
12	SP1	Shepparton	WSO. Surrounding inflammation	87 Male	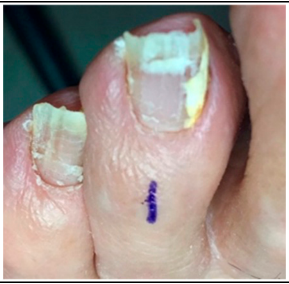
13	SP2	Shepparton	Advanced DLSO. Intact ventral surface. Dermatophytomas. Tinea pedis	55 Female	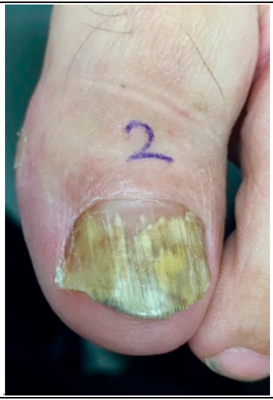

14	SP3	Shepparton	TDO. Multiple nail involvement. Tinea pedis	85 Female	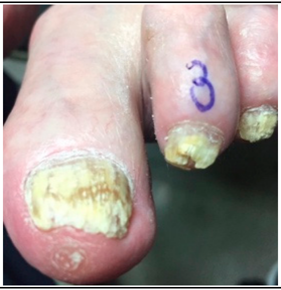
15	SP4	Shepparton	DLSO. Dermatophytomas. Multiple nails Tinea pedis	78 Female	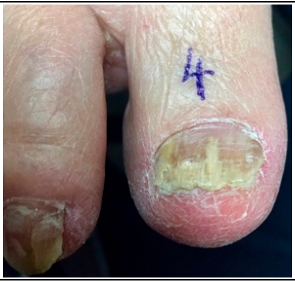
16	SP5	Shepparton	DLSO. Secondary nail SWO. Surrounding inflammation	73 Male	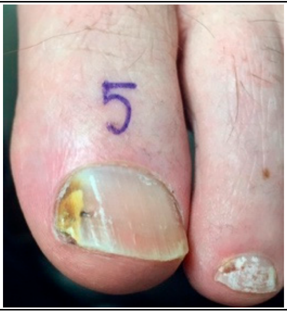
17	SP6	Shepparton	TDO Dermatophytomas. Cuticle severely infected. Tinea pedis	59 Male	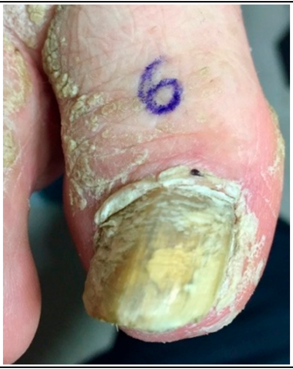
18	SP6b	Shepparton	DLSO. Dermatophytomas. Tinea pedis	72 Female	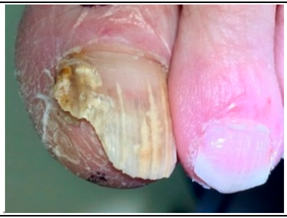
19	SP7	Shepparton	Great nail TDO. Small nail SWO. Tinea pedis	80 Male	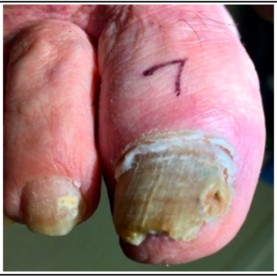

20	SPC (Control 2)	Shepparton	Skin inflammation medial side of toe. May be due to cornified lesion.	73 Female	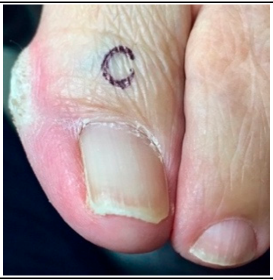
Summary	18 diseased 2 controls (Melbourne and Bass Coast) Total 20	4 Bass Coast 7 Melbourne 9 Shepparton		Mean age*: 79 years Gender. Female: 12 Male:7 Unknown 1	

Figure S1 Characteristics of samples used in the study. Nails were classified using the clinical appearance and classified as follows: DLSO: distal subungal onychomycosis. SWO: superficial white onychomycosis. TDO: total dystrophic onychomycosis**.** *When the exact age was unknown the midpoint in the decade was used for calculations e.g. 80s becomes 85. Numbers drawn on the toes were solely for the purpose of identification by the contributing podiatrist.

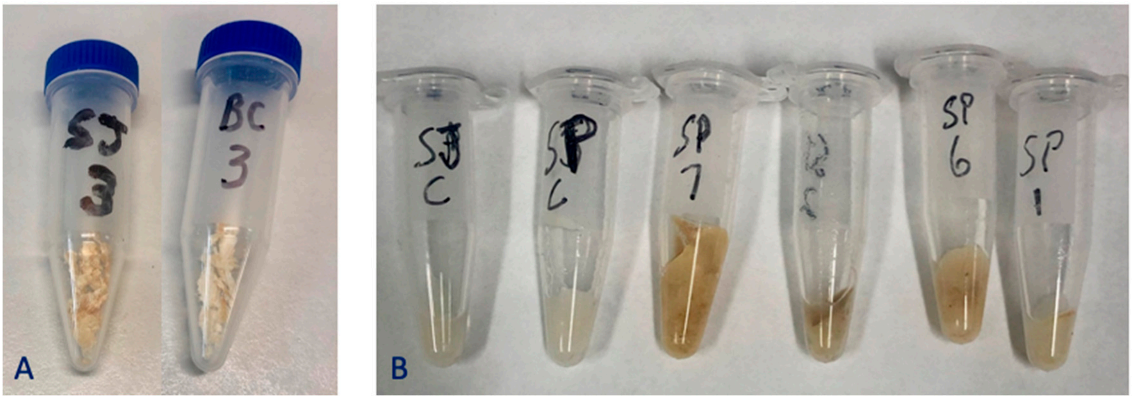

Figure S2 A Typical nail samples provided by podiatrists. Note the crumbling porous nature. **B** Samples after DNA extraction in 1.5 ml Eppendorf tubes. The two controls 11 (SJC) and 20 (SPC) show less pigmentation than infected nails.: 12 (SP1), 13 (SP2), 17 (SP6) and 19 (SP7)

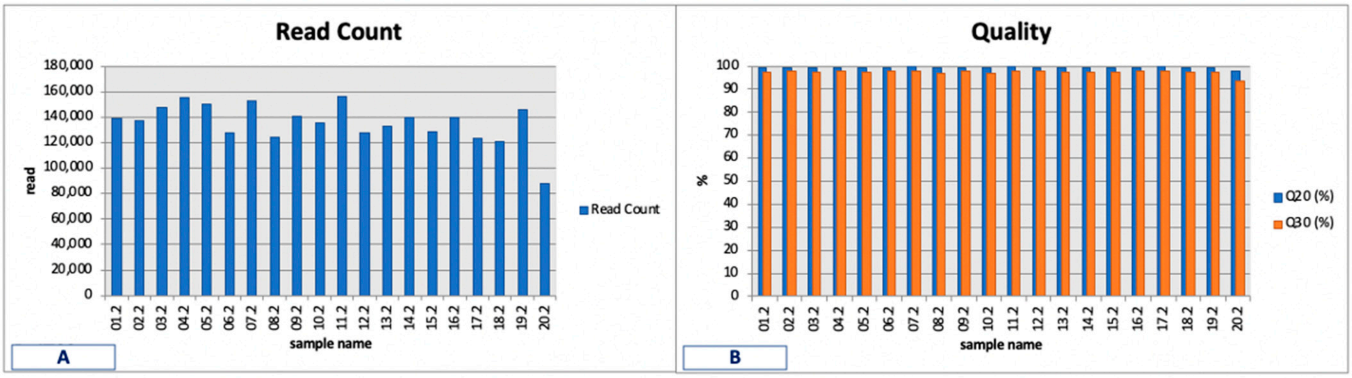

Figure S3 ITS2. A Read Counts **B** Phred scores**.** Sample read counts and sequence quality for ITS2 data. Read counts and Phred scores were all were all within the expected range (Reads 50-100K, Phred Q30>70-80%)

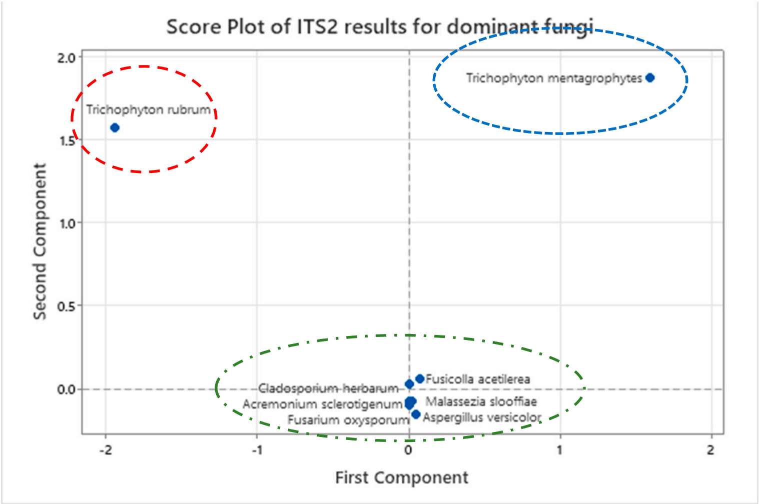

Figure S4 PCA score plot for high scoring fungi showing three clusters.

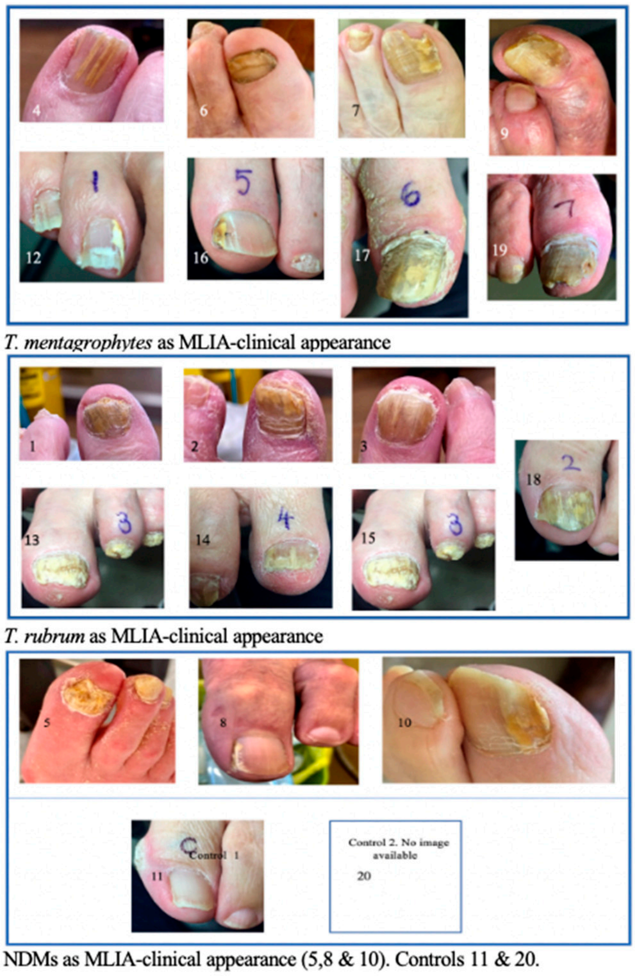

Figure S5 Clinical appearance of toenails grouped by MLIA. *Trichophyton mentagrophytes* (top), *T. rubrum* (middle) and an NDM (bottom) as the dominant fungus. Mycotic nails are 1-10 and 12-19; control nails are 11 and 20 (not pictured).

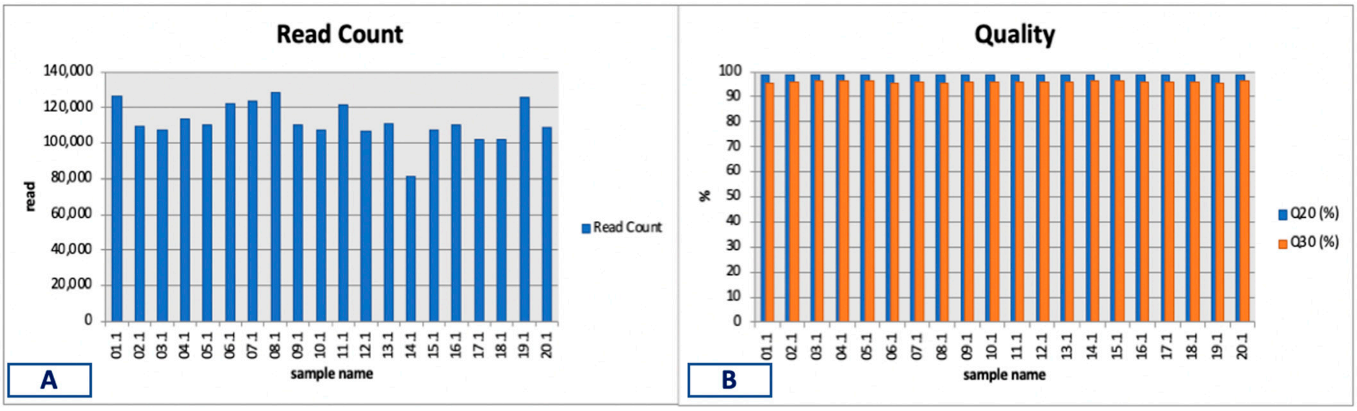

Figure S6 Sample read counts (A) and sequence quality (B) from 16S primers.

The top five results for the bacteria comprised well over 80% of the total population/ community detected in most nails. *Staphylococcus epidermidis* DNA was found in every nail including the two controls. *Rothia koreensis* was also prominent as was *Brevibacterium sediminis* (Table S5).

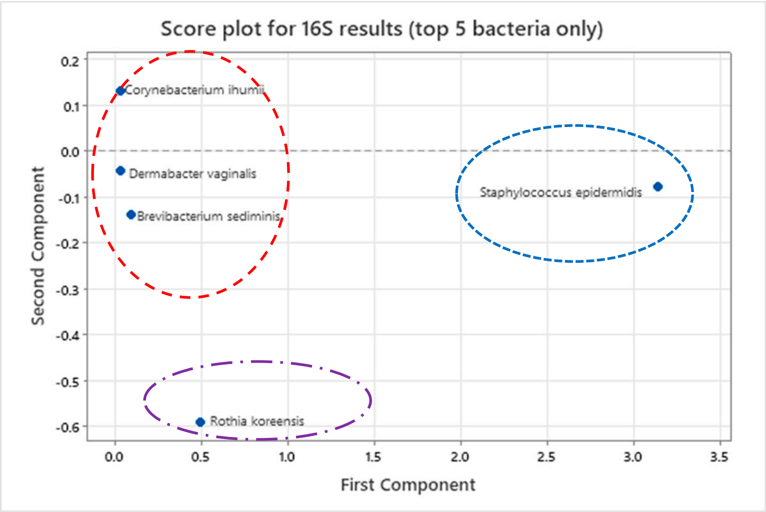

Figure S7 Score plot from PCA, showing the separation of three clusters of nails characterised by the subdominant bacteria**.**
