# Supplementary material for: Metagenomics of Toenail Onychomycosis in Three Victorian Regions of Australia
Source: J Fungi (Basel). 2022 Nov 14;8(11):1198. doi: 10.3390/jof8111198 (PMC9698484; doi:10.3390/jof8111198)
Supplement: Supplementary file 1 [file jof-08-01198-s001.zip › Supplementary Table S2 ITS2.pdf]

Supplementary Table S2. ITS2 rRNA Results

Nail 1

Nail 1. Species. ITS2	% Hits
Trichophyton rubrum	98.11
Trichophyton mentagrophytes	0.65
Filobasidium sp.	0.38
Cystobasidium sp.	0.22
Fungi	0.14
Candida parapsilosis	0.12
Naganishia diffluens	0.07
Meyerozyma guilliermondii	0.07
Aspergillus penicillioides	0.07
Cladosporium herbarum	0.05
Malassezia sp.	0.04
Cryptococcus uniguttulatus	0.03
Melissa officinalis	0.03
Total	100.00

Nail 2

Nail 2. Species. ITS2	% Hits
Trichophyton rubrum	95.53
Rhodotorula sp.	0.88
Candida parapsilosis	0.61
Aureobasidium pullulans	0.50
Verrucaria sp.	0.34
Unidentified	0.29
Malassezia sp.	0.25
Cladosporium herbarum	0.14
Epicoccum nigrum	0.10
Verrucariales	0.08
Fungi	0.08
Coniosporium sp.	0.07
Filobasidium sp.	0.06
Exobasidium vexans	0.06
Trebouxiophyceae	0.05
Cladosporium sphaerospermum	0.05

Capnodiaceae	0.05
Botrytis cinerea	0.05
Meyerozyma guilliermondii	0.04
Streptophyta sp.	0.04
Chaetothyriales	0.04
Scopulariopsis candida	0.04
Xenodidymella sp.	0.03
Cucurbitales	0.03
Neophaeococcomyces sp.	0.03
Rhodotorula babjevae	0.03
Zalaria obscura	0.03
Paraphoma sp.	0.03
Naganishia diffluens	0.03
Neocatenulostroma germanicum	0.03
Cystobasidium sp.	0.03
Pleosporales	0.02
Lecanicillium fungicola	0.02
Herpotrichiellaceae	0.02
Ascomycota	0.02
Phoma sp.	0.02
Curvularia coatesiae	0.02
Flavoplaca oasis	0.02
Bjerkandera sp.	0.01
Malasseziales	0.01
Arachis sp.	0.01
Triticum aestivum	0.01
Capnodiales	0.01
Yarrowia lipolytica	0.01
Beta sp.	0.01
Unassigned	0.01
Sporothrix sp.	0.01
Dianthus sp.	0.01
Thelebolus sp.	0.01
Verrucaria muralis	0.01
Physciaceae	0.01
Aspergillus versicolor	0.01
Alternaria infectoria	0.01
Ochroconis mirabilis	0.01
Dothideomycetes	0.01
Seiridium cardinale	0.01
Vishniacozyma victoriae	0.01
Trifolium glomeratum	0.01

Pseudotaeniolina globosa	0.01
Cladophialophora sp.	0.01
Kunzea baxteri	0.01
Chlorophyta	0.01
Miscellaneous unidentified	0.03
Total	100.00

Nail 3

**Nail 3. Species. ITS2**	**% Hits**
Trichophyton rubrum	99.93
Fungi	0.02
Hypholoma sp.	0.02
Trichophyton mentagrophytes	0.02
Debaryomyces hansenii	0.01
Total	100.00

Nail 4

**Nail 4. Species. ITS2**	**% Hits**
Trichophyton mentagrophytes	99.13
Epicoccum nigrum	0.10
Cladosporium herbarum	0.10
Exophiala eucalyptorum	0.09
Malassezia sp.	0.08
Filobasidium sp.	0.08
Vishniacozyma victoriae	0.06
Candida parapsilosis	0.02
Fungi	0.02
Sporidiobolus metaroseus	0.02
Unassigned	0.02
Holtermanniella sp.	0.02
Rhodotorula babjevae	0.01
Cladosporium sphaerospermum	0.01
Malasseziales	0.01
Rhodotorula sp.	0.01
Pyrenochaetopsis sp	0.01
Saccharomyces cerevisiae	0.01
Hortaea thailandica	0.01
Penicillium sp.	0.01

Trichophyton rubrum	0.01
Penicillium brevicompactum	0.01
Agaricus bisporus	0.01
Phaeosphaeria sp.	0.01
Liberomyces pistaciae	0.01
Dioszegia sp.	0.01
Verrucariales	0.01
Pleosporales	0.01
Fungi	0.01
Dissoconium aciculare	0.01
Helotiales	0.01
Microdochium seminicola	0.01
Cystofilobasidium capitatum	0.01
Melissa officinalis	0.01
Syzygium floribundum	0.01
Lecanicillium sp.	0.01
Streptophyta	0.01
Miscellaneous unidentified	0.05
Total	100.00

Nail 5

**Nail 5. Species. ITS2**	**% Hits**
Aspergillus versicolor	99.40
Cladosporium herbarum	0.09
Ascomycota	0.05
Epicoccum nigrum	0.03
Aureobasidium pullulans	0.03
Alternaria alternata	0.03
Solanum lycopersicum	0.03
Coprinellus velatopruinatus	0.03
Eukaryota kgd Incertae sedis	0.03
Neophaeococcomyces sp.	0.01
Verrucaria ahtii	0.01
Unassigned	0.01
Dothideomycetes	0.01
Auriculariales	0.01
Dothideomycetes	0.01
Vanderwaltozyma sp.	0.01
Xylodon sp.	0.01
Gaertneriomyces semiglobifer	0.01

Verrucariaceae	0.01
Neodevriesia sp.	0.01
Erysiphe alphitoides	0.01
Cyberlindnera sp.	0.01
Saccharomyces cerevisiae	0.01
Fungi	0.01
Toxicocladosporium rubrigenum	0.01
Chaetothyriales	0.01
Pichia fermentans	0.01
Ganoderma sp.	0.01
Peniophora lycii	0.01
Apiotrichum sp.	0.01
Botrytis cinerea	0.01
Kazachstania servazzii	0.01
Miscellaneous unidentified	0.10
Total	100.00

Nail 6

**Nail 6. Species. ITS2**	**% Hits**
Trichophyton mentagrophytes	70.39
Nigrospora oryzae	6.18
Epicoccum nigrum	6.11
Cladosporium herbarum	4.81
Ascomycota	3.86
Rhodotorula sp.	1.21
Cryptococcus uniguttulatus	0.72
Alternaria alternata	0.71
Aureobasidium pullulans	0.59
Pleosporales	0.36
Yarrowia lipolytica	0.31
Fungi	0.30
Vishniacozyma victoriae	0.25
Eukaryota Incertae sedis	0.23
Periconia sp.	0.22
Cladosporium sphaerospermum	0.19
Curvularia coatesiae	0.18
Unassigned	0.18
Tausonia pullulans	0.16
Pyrenophora erythrospila	0.16
Filobasidium sp.	0.15

Phaeosphaeria	0.10
Oidium sp.	0.10
Candida parapsilosis	0.09
Bipolaris sp.	0.09
Botrytis cinerea	0.09
Dothideomycetes	0.08
Phaeosphaeria sp.	0.07
Symmetrospora foliicola	0.06
Saitozyma sp.	0.05
Dothidea sp.	0.05
Pyrenophora sp.	0.05
Meyerozyma guilliermondii	0.05
Papiliotrema flavescens	0.04
Naganishia diffluens	0.04
Pichia fermentans	0.04
Melaleuca bracteata	0.04
Streptophyta	0.04
Bisifusarium dimerum	0.04
Rhynchogastrema tunnelae	0.04
Phaeothecoidea minutispora	0.04
Candida sp.	0.04
Goffeauzyma agrionensis	0.04
Saitozyma flava	0.04
Tetracladium marchalianum	0.04
Dothioraceae	0.03
Pseudosydowia eucalypti	0.03
Verrucariaceae	0.03
Curvibasidium pallidicorallinum	0.03
Fonsecazyma sp.	0.03
Chaetothyriales	0.03
Dothiorella sarmentorum	0.03
Curvularia kusanoi	0.03
Aspergillus fumigatus	0.02
Phaeosphaeriaceae	0.02
Alternaria infectoria	0.02
Erythrobasidium sp.	0.02
Malassezia sp.	0.02
Pilidium concavum	0.02
Capnodiales	0.02
Coniella sp.	0.02
Rhodotorula babjevae	0.02
Talaromyces sp.	0.02

Xenodidymella sp.	0.02
Cryptovalsa ampelina	0.02
Vishniacozyma heimaeyensis	0.02
Penicillium sp.	0.02
Sporidiobolaceae	0.02
Vishniacozyma dimennae	0.02
Chaetothyriales	0.02
Geopyxis sp.	0.02
Tremellales	0.02
Jasminum elongatum	0.02
Keissleriella cladophila	0.01
Malassezia slooffiae	0.01
Paraconiothyrium sp.	0.01
Fungi	0.01
Coniosporium sp.	0.01
Cytospora austromontana	0.01
Buckleyzyma aurantiaca	0.01
Papiliotrema frias	0.01
Stemphylium sp.	0.01
Phaeotheca sp.	0.01
Vanderwaltozyma sp.	0.01
Alfaria putrefolia	0.01
Septoria sp.	0.01
Exophiala eucalyptorum	0.01
Rosa banksiae	0.01
Dothideomycetes sp	0.01
Phomatospora dinemasporium	0.01
Eucalyptus botryoides	0.01
Neophaeococcomyces sp.	0.01
Phacidiella eucalypti	0.01
Ascomycota	0.01
Buckleyzyma armeniaca	0.01
Pseudorobillarda sp.	0.01
Aspergillus sp.	0.01
Colletotrichum sp.	0.01
Clonostachys sp.	0.01
Microdochium seminicola	0.01
Cystobasidium sp.	0.01
Ustilago sp.	0.01
Didymellaceae	0.01
Kockovaella sp.	0.01
Didymella sp.	0.01

Ramularia plurivora	0.01
Aspergillus niger	0.01
Diplocarpon rosae	0.01
Phaeosphaeriaceae	0.01
Candida albicans	0.01
Verrucariales	0.01
Phacidium sp.	0.01
Pyrenochaetopsis sp.	0.01
Cylindrodendrum sp.	0.01
Paraphaeosphaeria michotii	0.01
Pleurophoma sp.	0.01
Thyridariaceae	0.01
Pleosporales	0.01
Monocillium tenue	0.01
Cystobasidium lysinophilum	0.01
Spencerozyma crocea	0.01
Rhodosporidiobolus colostri	0.01
Naganishia sp.	0.01
Cryptococcus sp.	0.01
Fraxinus angustifolia	0.01
Prunus sp.	0.01
Trametes sp.	0.01
Sympoventuria capensis	0.01
Penicillium glabrum	0.01
Phaeomoniellales	0.01
Debaryomyces hansenii	0.01
Fusarium chlamydosporum	0.01
Sordariomycetes	0.01
Basidiomycota	0.01
Vishniacozyma sp.	0.01
Lophiostoma corticola	0.01
Tetraplosphaeria sp.	0.01
Venturiaceae	0.01
Talaromyces ramulosus	0.01
Diaporthe foeniculina	0.01
Cytospora variostromatica	0.01
Cystofilobasidium infirmominiatum	0.01
Dioszegia sp.	0.01
Miscellaneous unidentified	0.13
Total	100.00

Nail 7

Nail 7. Species. ITS2	% Hits
Trichophyton mentagrophytes	99.45
Fusarium chlamydosporum	0.14
Candida parapsilosis	0.06
Malasseziales	0.05
Malassezia sp.	0.04
Farysia sp.	0.04
Coniosporium sp.	0.04
Penicillium sp.	0.04
Fungi	0.04
Cladosporium sphaerospermum	0.02
Alternaria alternata	0.02
Moringa oleifera	0.02
Trichophyton rubrum	0.02
Exophiala dermatitidis	0.01
Melissa officinalis	0.01
Total	100.00

Nail 8

Nail 8. Species. ITS2	% Hits
Acremonium sclerotigenum	86.27
Epicoccum nigrum	3.34
Cladosporium herbarum	1.75
Malassezia sp.	1.39
Filobasidium sp.	0.80
Rhodotorula sp.	0.58
Bjerkandera sp.	0.49
Cladosporium sphaerospermum	0.37
Fungi	0.32
Grammothele sp.	0.31
Rhodotorula babjevae	0.28
Unassigned	0.28
Vishniacozyma victoriae	0.27
Microbotryomycetes.	0.25
Aureobasidium pullulans	0.23
Candida parapsilosis	0.20
Curvularia lunata	0.17

Meyerozyma guilliermondii	0.16
Penicillium corylophilum	0.16
Cenchrus sp.	0.14
Aspergillus parasiticus	0.13
Cystobasidiomycetes	0.12
Yarrowia lipolytica	0.12
Scopulariopsis candida	0.11
Cystobasidium sp.	0.10
Neodevriesiaceae	0.10
Mycocalicium victoriae	0.10
Exophiala sp.	0.09
Malassezia slooffiae	0.09
Acarosporales	0.09
Oxalis corniculata	0.08
Moringa oleifera	0.08
Naganishia diffluens	0.07
Rutstroemia sp.	0.07
Eukaryota kgd Incertae sedis	0.07
Penicillium decumbens	0.07
Malassezia sympodialis	0.07
Trifolium glomeratum	0.06
Exophiala mesophila	0.06
Botrytis cinerea	0.06
Peniophora lycii	0.06
Eugenia biflora	0.06
Cutaneotrichosporon mucoides	0.06
Verrucariaceae	0.05
Linum sp.	0.05
Phaeosphaeria sp.	0.05
Phaeosphaeria sp.	0.05
Pisum sativum	0.04
Penicillium sp.	0.03
Podosphaera erodii	0.02
Chaetothyriales	0.01
Orbiliales	0.01
Streptophyta	0.01
Total	100.00

Nail 9

**Nail 9. Species. ITS2**	**Hits**

Trichophyton mentagrophytes	90.23
Malasseziales	1.34
Malassezia sp.	1.22
Malassezia sp.	0.68
Pseudocercosporella fraxini	0.56
Fungi	0.51
Cladosporium sphaerospermum	0.50
Filobasidium sp.	0.40
Aureobasidium pullulans	0.36
Cladosporium herbarum	0.33
Incertae sediss unidentified	0.30
Acremonium sp.	0.29
Epicoccum nigrum	0.26
Pisum sativum	0.23
Rhodotorula sp.	0.21
Alternaria alternata	0.19
Cutaneotrichosporon mucoides	0.14
Coniosporium sp.	0.13
Unassigned	0.12
Meira sp.	0.11
Fungi	0.11
Rhizoplaca polymorpha	0.10
Eugenia biflora	0.10
Rhizoplaca subdiscrepans	0.09
Naganishia diffluens	0.09
Linum	0.08
Ramularia plurivora	0.08
Musa sp.	0.07
Neophaeococcomyces sp.	0.07
Lagerstroemia	0.06
Verrucaria macrostoma	0.05
Acremonium citrinum	0.05
Verrucariaceae	0.05
Acremonium sclerotigenum	0.05
Malassezia slooffiae	0.05
Melissa officinalis	0.04
Verrucaria nigrescens	0.04
Gibberella acuminata	0.04
Ascomycota	0.04
Triticum aestivum	0.04
Moringa oleifera	0.03
Chlorophyta	0.03

Sarcogyne regularis	0.03
Vishniacozyma victoriae	0.03
Dothideomycetes	0.03
Fusarium chlamydosporum	0.03
Streptophyta	0.03
Candida parapsilosis	0.03
Cladosporium sp.	0.03
Rhodotorula babjevae	0.03
Pyrenochaeta unguis-hominis	0.02
Verrucaria muralis	0.02
Leptospermum lanigerum	0.02
Saccharomyces cerevisiae	0.02
Corymbia citriodora	0.02
Phaeomoniellales	0.02
Sarocladium sp.	0.02
Phlebiporia bubalina	0.02
Acarosporales	0.02
Cucurbitales	0.01
Trebouxia sp.	0.01
Penicillium sp.	0.01
Cystofilobasidium capitatum	0.01
Pycnoporus coccineus	0.01
Debaryomyces hansenii	0.01
Camellia semiserrata	0.01
Penicillium steckii	0.01
Platanus sp.	0.01
Miscellaneous unidentified	0.02
Total	100.00

Nail 10

**Nail 10. Species. ITS2**	**% Hits**
Eukaryota kgd Incertae sedis/*Fusarium oxysporum*	86.27
Trichophyton mentagrophytes	12.81
Malassezia sp.	0.49
Rhodotorula sp.	0.08
Pseudocercosporella fraxini	0.07
Cladosporium herbarum	0.07
Hyphoderma subsetigerum	0.04
Botryobasidium sp.	0.03
Xylodon sp.	0.03

Candida glabrata	0.03
Candida parapsilosis	0.02
Malassezia sympodialis	0.02
Melissa officinalis	0.01
Trichophyton rubrum	0.01
Malasseziales	0.01
Miscellaneous unidentified	0.01
Total	100.00

Nail 11

**Nail 11 (Control 1).Species. ITS2**	**% Hits**
Eukaryota kgd Incertae sedis/*Fusarium oxysporum*	95.77
Epicoccum nigrum	1.13
Aspergillus versicolor	0.90
Malassezia sp.	0.43
Periconia macrospinosa	0.39
Cladosporium herbarum	0.30
Ascomycota	0.18
Candida parapsilosis	0.16
Melissa officinalis	0.10
Trichophyton rubrum	0.08
Candida albicans	0.07
Capnodiales	0.06
Trichophyton mentagrophytes	0.06
Pyrenophora sp.	0.04
Alternaria infectoria	0.04
Naganishia diffluens	0.04
Unassigned	0.03
Malassezia sympodialis	0.03
Sistotrema sp.	0.02
Ochroconis mirabilis	0.02
Fungi	0.02
Botrytis cinerea	0.02
Ascomycota	0.02
Cystobasidium laryngis	0.02
Acer saccharinum	0.02
Penicillium dierckxii	0.02
Pichia fermentans	0.01
Penicillium brevicompactum	0.01
Miscellaneous unidentified	0.01

Total	100.00

Nail 12

**Nail 12. Species. ITS2**	**Hits**
Trichophyton mentagrophytes	94.73
Filobasidium sp.	1.39
Malassezia sp.	0.65
Alternaria alternata	0.57
Cladosporium herbarum	0.49
Naganishia diffluens	0.38
Malassezia slooffiae	0.30
Vishniacozyma victoriae	0.27
Rhodotorula sp.	0.18
Papiliotrema frias	0.17
Epicoccum nigrum	0.12
Melissa officinalis	0.10
Saccharomyces cerevisiae	0.08
Botrytis cinerea	0.06
Pichia sp.	0.05
Sigarispora sp.	0.05
Debaryomyces hansenii	0.04
Penicillium sp.	0.04
Vishniacozyma dimennae	0.04
Tausonia pullulans	0.03
Pseudozyma sp.	0.02
Meyerozyma guilliermondii	0.02
Candida parapsilosis	0.02
Trichophyton rubrum	0.02
Fungi	0.02
Pichia membranifaciens	0.02
Capnodiales	0.02
Pseudotaeniolina globosa	0.01
Malasseziales	0.01
Streptophyta	0.01
Basidiomycota	0.01
Rhizopus arrhizus	0.01
Lophostemon confertus	0.01
Fungi	0.01
Helotiales	0.01
Cladosporium sphaerospermum	0.01

Trebouxia sp.	0.01
Zymoseptoria tritici	0.01
Microbotryomycetes	0.01
Unidentified	0.01
Total	100.00

Nail 13

Nail 13. Species. ITS2	% Hits
Trichophyton rubrum	94.82
Streptophyta	2.21
Cladosporium herbarum	0.88
Alternaria sp.	0.32
Malassezia sp.	0.21
Holtermanniella sp.	0.17
Melaleuca bracteata	0.16
Candida parapsilosis	0.13
Epicoccum nigrum	0.09
Coniella sp.	0.08
Saccharomyces cerevisiae	0.06
Melissa officinalis	0.06
Lectera colletotrichoides	0.05
Sporormiaceae	0.05
Fungi	0.05
Unassigned	0.04
Alternaria infectoria	0.04
Alternaria alternata	0.04
Fusarium chlamydosporum	0.04
Fusicolla acetilerea	0.04
Dimorphiseta acuta	0.04
Alfaria putrefolia	0.03
Coniosporium sp.	0.03
Pseudocercosporella fraxini	0.03
Rhodosporidiobolus colostri	0.03
Aspergillus sp.	0.02
Penicillium sp.	0.02
Toxicocladosporium rubrigenum	0.02
Microbotryum sp.	0.02
Melaleuca linariifolia	0.02
Lactuca sativa	0.02
Teratosphaeriaceae	0.02

Gymnoascus sp.	0.01
Allium sp.	0.01
Triticum aestivum	0.01
Lolium sp.	0.01
Aspergillus sparsus	0.01
Malva parviflora	0.01
Arctotheca sp.	0.01
Salvia sp.	0.01
Dothideales	0.01
Solanum lycopersicum	0.01
Prunus sp.	0.01
Miscellaneous unidentified	0.02
Total	100.00

Nail 14

**Nail 14. Species. ITS2**	**% Hits**
Trichophyton rubrum	98.62
Cladosporium herbarum	0.43
Alternaria sp.	0.19
Epicoccum nigrum	0.16
Alternaria alternata	0.12
Ascomycota	0.07
Rhodotorula sp.	0.05
Fungi	0.04
Candida parapsilosis	0.03
Sporidiobolus metaroseus	0.03
Golovinomyces orontii	0.02
Neoascochyta cylindrispora	0.02
Pleosporales	0.02
Ochroconis mirabilis	0.02
Podosphaera erodii	0.02
Rhodotorula babjevae	0.02
Unassigned	0.01
Pseudocercosporella fraxini	0.01
Coprinellus micaceus	0.01
Pseudozyma sp.	0.01
Hymenochaetales	0.01
Penicillium sp.	0.01
Helotiales sp.	0.01
Melissa officinalis	0.01

Symmetrospora foliicola	0.01
Dothideomycetes	0.01
Eukaryota Incertae sedis	0.01
Miscellaneous unidentified	0.03
Total	100.00

Nail 15

**Nail 15. Species. ITS2**	**% Hits**
Trichophyton rubrum	97.77
Cladosporium herbarum	0.72
Aureobasidium pullulans	0.22
Agaricus bisporus	0.20
Epicoccum nigrum	0.17
Fungi	0.07
Alternaria alternata	0.06
Malassezia sp.	0.05
Pleosporales	0.05
Naganishia diffluens	0.05
Alternaria sp.	0.04
Ascomycota	0.04
Candida parapsilosis	0.04
Vishniacozyma victoriae	0.04
Ustilago sp.	0.04
Phialemonium inflatum	0.03
Cystobasidium sp.	0.03
Rhodotorula babjevae	0.03
Botrytis cinerea	0.03
Eukaryota Incertae sedis	0.03
Alternaria infectoria	0.03
Filobasidium sp.	0.02
Fusarium chlamydosporum	0.02
Holtermanniella sp.	0.02
Neoascochyta cylindrispora	0.02
Unassigned	0.02
Ceratobasidiaceae	0.02
Scedosporium aurantiacum	0.01
Vishniacozyma dimennae	0.01
Verrucariales	0.01
Streptophyta	0.01
Pleosporales	0.01

Symmetrospora foliicola	0.01
Xanthocarpia sp.	0.01
Cladosporium sphaerospermum	0.01
Moringa oleifera	0.01
Rosa sp.	0.01
Amyema sp.	0.01
Papiliotrema frias	0.01
Miscellaneous unidentified	0.04
Total	100.00

Nail 16

**Nail 16. Species. ITS2**	**% Hits**
Trichophyton mentagrophytes	90.43
Trifolium glomeratum	2.40
Aureobasidium pullulans	1.46
Epicoccum nigrum	0.91
Tremella sp.	0.73
Fungi	0.62
Cladosporium herbarum	0.61
Meyerozyma guilliermondii	0.49
Malassezia sp.	0.37
Rhodotorula sp.	0.28
Exophiala eucalyptorum	0.23
Penicillium sp.	0.16
Penicillium brevicompactum	0.15
Candida parapsilosis	0.10
Erythrobasidium sp.	0.10
Alternaria alternata	0.07
Ustilago cynodontis	0.07
Unassigned	0.06
Pseudocercosporella fraxini	0.06
Eukaryota Incertae sedis	0.06
Agaricomycetes	0.06
Filobasidium sp.	0.05
Parafenestella rosacearum	0.05
Periconiaceae	0.05
Penicillium glabrum	0.05
Rhodotorula babjevae	0.04
Pleosporales	0.04
Helotiales	0.04

Naganishia sp.	0.04
Streptophyta	0.03
Brachysporium nigrum	0.03
Ascomycota	0.03
Papiliotrema frias	0.03
Debaryomyces hansenii	0.02
Hormonema sp.	0.02
Pseudozyma sp.	0.02
Conocybe fuscimarginata	0.02
Dothideomycetes	0.01
Onygenales Incertae sedis	0.01
Wardomycopsis humicola	0.01
19artalini asp.	0.01
Argyranthemum sp.	0.01
Vishniacozyma victoriae	0.01
Papaver sp.	0.01
Miscellaneous unidentified	0.01
Total	100.00

Nail 17

**Nail 17. Fungi. ITS2**	**% Hits**
Trichophyton mentagrophytes	97.63
Cladosporium herbarum	0.85
Candida parapsilosis	0.35
Alternaria alternata	0.33
Ustilago cynodontis	0.29
Malassezia sp.	0.13
Fusarium chlamydosporum	0.06
Epicoccum nigrum	0.05
Dothideomycetes	0.05
Pseudocercosporella fraxini	0.04
Fungi	0.03
Eukaryota Incertae sedis	0.03
Cylindrium sp.	0.03
Capnodiales sp.	0.03
Melissa officinalis	0.02
Alternaria infectoria	0.02
Saccharomyces cerevisiae	0.02
Ascomycota	0.02

Eucalyptus botryoides	0.01
Aspergillus ochraceus	0.01
Total	100.00

Nail 18

**Nail 18. Species. ITS2**	**% Hits**
Trichophyton rubrum	83.80
Cladosporium herbarum	2.86
Malassezia sp.	2.19
Epicoccum nigrum	1.49
Alternaria alternata	0.92
Filobasidium sp.	0.73
Alternaria sp.	0.56
Malasseziales	0.47
Rhodotorula sp.	0.44
Fungi	0.39
Stemphylium sp.	0.36
Stachybotrys chartarum	0.34
Ascomycota	0.33
Penicillium sp.	0.33
Aureobasidium pullulans	0.32
Pleosporales	0.25
Trebouxia sp.	0.24
Alternaria infectoria	0.22
Debaryomyces hansenii	0.21
Eukaryota Incertae sedis	0.19
Fusarium chlamydosporum	0.18
Naganishia diffluens	0.16
Candida parapsilosis	0.13
Rhizophlyctis sp.	0.12
Solanum lycopersicum	0.12
Malassezia globosa	0.12
Arctotheca sp.	0.09
Gibberella acuminata	0.09
Mucor racemosus	0.09
Botrytis cinerea	0.09
Erodium sp.	0.08
Pseudocercosporella fraxini	0.08
Coniosporium sp	0.08
Rhodotorula babjevae	0.07

Cryptococcus uniguttulatus	0.07
Saccharomyces cerevisiae	0.07
Tausonia pullulans	0.07
Cladosporium sphaerospermum	0.07
Dothideomycetes	0
Exophiala xenobiotica	0.06
Brassica rapa subsp. *oleifera*	0.06
Pleosporales	0.06
Eucalyptus botryoides	0.05
Holtermanniella sp..	0.05
Eucalyptus botryoides	0.05
Malassezia sympodialis	0.05
Holtermanniella sp.	0.05
Exophiala eucalyptorum	0.04
Vishniacozyma sp.	0.04
Meyerozyma guilliermondii	0.04
Botryotrichum murorum	0.04
Unassigned	0.04
Leratiomyces ceres	0.04
Leucosporidium sp.	0.04
Yarrowia lipolytica	0.04
Chaetothyriales	0.03
Phialemonium inflatum	0.03
Metarhizium marquandii	0.03
Papiliotrema frias	0.03
Callistemon viminalis	0.03
Anthophyta	0.03
Vishniacozyma victoriae	0.02
Herpotrichiellaceae	0.02
Acer negundo	0.02
Basidiobolus ranarum	0.02
Fraxinus angustifolia	0.02
Ceratobasidiaceae	0.02
Podosphaera erodii	0.02
Arthrobotrys	0.02
Leotiomycetes	0.02
Pseudozyma sp.	0.02
Trichoderma sp.	0.02
Dimorphiseta acuta	0.02
Cryptococcus laurentii	0.01
Trichosporon aquatile	0.01
Issatchenkia orientalis	0.01

Udeniomyces puniceus	0.01
Penicillium brevicompactum	0.01
Cytospora sp.	0.01
Lectera colletotrichoides	0.01
Olea europaea subsp. europaea	0.01
Phaeococcomyces sp.	0.01
Coelastrella sp.	0.01
Phaeosphaeria sp.	0.01
Lolium sp.	0.01
Cystobasidium sp.	0.01
Melaleuca bracteata	0.01
Chaetothyriales	0.01
Lycoperdon rupicola	0.01
Mortierella sp.	0.01
Hymenochaete sp.	0.01
Aspergillus versicolor	0.01
Persicaria capitata	0.01
Malva parviflora	0.01
Platanus sp.	0.01
Pyrus salicifolia	0.01
Triticum aestivum	0.01
Helianthus sp.	0.01
Hanseniaspora uvarum	0.01
Cucumis sp.	0.01
Callistemon salignus	0.01
Miscellaneous unidentified	0.04
Total	100.00

Nail 19

**Nail 19. Species. ITS2**	**% Hits**
Fusicolla acetilerea	51.92
Trichophyton mentagrophytes	47.55
Epicoccum nigrum	0.18
Alternaria alternata	0.15
Cladosporium herbarum	0.11
Cytospora eucalypticola	0.02
Candida parapsilosis	0.02
Cladosporium sphaerospermum	0.01
Filobasidium sp.	0.01
Fusarium chlamydosporum	0.01

Alternaria sp.	0.01
Fungi	0.01
Miscellaneous unidentified	0.01
Total	100.00

Nail 20

**Nail 20. (Control 2). Species. ITS2**	**% Hits**
Malassezia slooffiae	93.57
Trichophyton mentagrophytes	3.89
Cladosporium herbarum	0.97
Epicoccum nigrum	0.20
Ascomycota	0.14
Fungi	0.13
Alternaria alternata	0.11
Pleosporales	0.09
Alternaria sp.	0.07
Malassezia sp.	0.07
Aureobasidium pullulans	0.06
Eukaryota kgd Incertae sedis	0.05
Phaeosphaeria sp.	0.05
Phaeosphaeria sp.	0.05
Rhodotorula sp.	0.05
Phaeosphaeriaceae sp.	0.03
Naganishia diffluens	0.03
Periconia sp.	0.03
Paraphaeosphaeria michotii	0.03
Plantago coronopus	0.02
Candida parapsilosis	0.02
Unassigned	0.02
Xylodon sp.	0.02
Toxicocladosporium rubrigenum	0.02
Albifimbria verrucaria	0.01
Nigrospora sp.	0.01
Vishniacozyma victoriae	0.01
Bartalinia sp.	0.01
Dothideomycetes	0.01
Penicillium sp.	0.01
Truncatella restionacearum	0.01
Fuscoporia contigua	0.01
Fibroporia vaillantii	0.01

Sistotremastrum sp.	0.01
Pseudocercosporella fraxini	0.01
Hymenochaetales	0.01
Fungi	0.01
Naganishia sp.	0.01
Petrophila sp.	0.01
Fusarium chlamydosporum	0.01
Rhizoctonia sp.	0.01
Fomitopsis lilacinogilva	0.01
Phlebiporia bubalina	0.01
Cladosporium sphaerospermum	0.01
Stemphylium sp.	0.01
Hermatomyces sp.	0.01
Aspergillus versicolor	0.01
Perenniporia maackiae	0.01
Dothideomycetes	0.01
Sakaguchia dacryoidea	0.01
Leucosporidium sp	0.01
Cryptococcus uniguttulatus	0.01
Miscellaneous unidentified	0.04
Total	100.00
