# Supplementary material for: Metagenomics of Toenail Onychomycosis in Three Victorian Regions of Australia
Source: J Fungi (Basel). 2022 Nov 14;8(11):1198. doi: 10.3390/jof8111198 (PMC9698484; doi:10.3390/jof8111198)
Supplement: Supplementary file 1 [file jof-08-01198-s001.zip › Supplementary Table S3 16S docx.pdf]

Supplementary Table S3 16S rRNA results

Nail 1

**Nail 1. 16S**	**Hits %**
Staphylococcus epidermidis	36.96
Rothia koreensis	19.83
Dermabacter vaginalis	13.39
Brevibacterium sediminis	11.86
Corynebacterium glyciniphilum	8.97
Brevibacterium paucivorans	6.62
Dietzia timorensis	0.90
Corynebacterium resistens	0.84
Corynebacterium tuberculostearicum	0.30
Propioniferax innocua	0.16
Brachybacterium faecium	0.05
Escherichia fergusonii	0.03
Clavibacter michiganensis	0.03
Corynebacterium jeikeium	0.01
Kocuria uropygioeca	0.01
Corynebacterium massiliense	0.01
Miscellaneous unidentified	0.03
Total	100.00

Nail 2

**Nail 2**	**Hits**
Staphylococcus epidermidis	61.79
Rothia koreensis	21.47
Brevibacterium sediminis	16.12
Enterococcus hirae	0.21
Corynebacterium tuberculostearicum	0.13
Dermabacter vaginalis	0.10
Dietzia timorensis	0.04
Kocuria uropygioeca	0.04
Staphylococcus argensis	0.03
Ignavigranum ruoffiae	0.02
Corynebacterium amycolatum	0.01
Staphylococcus simulans	0.01
Corynebacterium massiliense	0.01
Mucilaginibacter gotjawali	0.01
Moraxella osloensis	0.01

Brachybacterium faecium	0.01
Staphylococcus lugdunensis	0.01
Miscellaneous unidentified	0.01
Total	100.00

Nail 3

**Nail 3 16S**	**Hits %**
Staphylococcus epidermidis	99.80
Cutibacterium acnes	0.07
Corynebacterium tuberculostearicum	0.04
Brevibacterium sediminis	0.02
Corynebacterium falsenii	0.01
Corynebacterium ihumii	0.01
Corynebacterium massiliense	0.01
Brevibacterium paucivorans	0.01
Miscellaneous unidentified	0.03
Total	100.00

Nail 4

**Nail 4 16S**	**Hits %**
Staphylococcus epidermidis	67.99
Rothia koreensis	24.32
Brevibacterium sediminis	7.18
Corynebacterium tuberculostearicum	0.17
Dietzia timorensis	0.11
Micrococcus luteus	0.07
Brevundimonas albigilva	0.04
Moraxella osloensis	0.03
Kocuria uropygioeca	0.02
Corynebacterium ihumii	0.01
Staphylococcus argensis	0.01
Corynebacterium bovis	0.01
Staphylococcus lugdunensis	0.01
Acinetobacter tjernbergiae	0.01
Miscellaneous unidentified	0.02
Total	100.00

Nail 5

**Nail 5 16S**	**Hits %**
Staphylococcus epidermidis	95.52

Rothia koreensis	1.89
Corynebacterium tuberculostearicum	1.02
Corynebacterium massiliense	0.32
Dermabacter vaginalis	0.31
Anaerococcus senegalensis	0.31
Corynebacterium falsenii	0.18
Anaerococcus octavius	0.04
Nosocomiicoccus massiliensis	0.04
Streptococcus periodonticum	0.04
Corynebacterium ihumii	0.03
Pseudoglutamicibacter cumminsii	0.02
Propioniferax innocua	0.02
Peptoniphilus olsenii	0.02
Corynebacterium urealyticum	0.02
Finegoldia magna	0.02
Corynebacterium frankenforstense	0.02
Brevibacterium paucivorans	0.02
Porphyromonas pasteri	0.02
Peptoniphilus ivorii	0.02
Aerococcus urinae	0.01
Peptoniphilus grossensis	0.01
Leptotrichia wadei	0.01
Corynebacterium pyruviciproducens	0.01
Gemmiger formicilis	0.01
Anaerococcus urinomassiliensis	0.01
Neisseria elongata	0.01
Corynebacterium bovis	0.01
Corynebacterium riegelii	0.01
Brevibacterium sediminis	0.01
Bacteroides ovatus	0.01
Staphylococcus argensis	0.01
Staphylococcus simulans	0.01
Blautia wexlerae	0.01
Erwinia persicina	0.01
Unidentified	0.01
Miscellaneous unidentified	0.03
Total	100.00

Nail 6

**Nail 6 16S**	**Hits %**
Staphylococcus epidermidis	36.70

Corynebacterium ihumii	32.73
Corynebacterium tuberculostearicum	8.47
Anaerococcus octavius	4.53
Anaerococcus urinomassiliensis	2.58
Finegoldia magna	2.21
Dermabacter vaginalis	2.08
Facklamia languida	1.48
Anaerococcus provencensis	1.40
Alloiococcus otitis	1.19
Salinicoccus salitudinis	1.19
Peptoniphilus ivorii	1.07
Corynebacterium jeikeium	0.79
Helcococcus kunzii	0.67
Psychrobacter faecalis	0.45
Peptoniphilus olsenii	0.41
Corynebacterium frankenforstense	0.39
Anaerococcus senegalensis	0.33
Peptoniphilus obesi	0.17
Micrococcus luteus	0.17
Eremococcus coleocola	0.12
Nesterenkonia halophila	0.12
Propioniferax innocua	0.11
Ignavigranum ruoffiae	0.11
Jeotgalicoccus huakuii	0.10
Atopostipes suicloacalis	0.09
Salinicoccus qingdaonensis	0.05
Moraxella osloensis	0.04
Brevibacterium sediminis	0.04
Brevibacterium paucivorans	0.03
Clavibacter michiganensis	0.03
Rothia koreensis	0.03
Sinomonas flava	0.03
Staphylococcus argensis	0.02
Aliicoccus persicus	0.02
Corynebacterium bovis	0.01
Brachybacterium faecium	0.01
Kytococcus sedentarius	0.01
Corynebacterium massiliense	0.01
Dietzia timorensis	0.01
Rubrobacter xylanophilus	0.01
Faecalibacterium prausnitzii	0.01
Miscellaneous unidentified	0.02

Total	100.00

Nail 7

**Nail 7 16S**	**Hits %**
Staphylococcus epidermidis	89.85
Rothia koreensis	8.47
Acinetobacter tjernbergiae	0.63
Acinetobacter guillouiae	0.45
Moraxella osloensis	0.16
Cutibacterium acnes	0.10
Corynebacterium tuberculostearicum	0.09
Escherichia fergusonii	0.04
Rubrobacter xylanophilus	0.04
Corynebacterium resistens	0.02
Corynebacterium jeikeium	0.02
Corynebacterium ihumii	0.01
Clavibacter michiganensis	0.01
Corynebacterium massiliense	0.01
Brevibacterium sediminis	0.01
Corynebacterium bovis	0.01
Corynebacterium amycolatum	0.01
Acinetobacter ursingii	0.01
Brevibacterium paucivorans	0.01
Faecalibacterium prausnitzii	0.01
Anaerococcus octavius	0.01
Miscellaneous unidentified	0.04
Total	100.00

Nail 8

**Nail 8 16S**	**Hits %**
Staphylococcus epidermidis	39.50
Brevibacterium sediminis	16.86
Corynebacterium tuberculostearicum	16.83
Rothia koreensis	13.95
Corynebacterium jeikeium	4.54
Dermabacter vaginalis	2.44
Brevibacterium paucivorans	1.68
Dietzia timorensis	0.96
Corynebacterium massiliense	0.87
Corynebacterium resistens	0.64

Anaerococcus octavius	0.51
Propioniferax innocua	0.36
Corynebacterium ihumii	0.28
Streptomyces eurythermus	0.28
Clavibacter michiganensis	0.06
Facklamia languida	0.05
Brachybacterium faecium	0.04
Anaerococcus nagyae	0.04
Kocuria uropygioeca	0.02
Corynebacterium frankenforstense	0.02
Corynebacterium falsenii	0.01
Brevibacterium luteolum	0.01
Peptoniphilus ivorii	0.01
Corynebacterium bovis	0.01
Eremococcus coleocola	0.01
Ignavigranum ruoffiae	0.01
Finegoldia magna	0.01
Miscellaneous unidentified	0.02
Total	100.00

Nail 9

**Nail 9 16S**	**Hits %**
Staphylococcus epidermidis	66.32
Rothia koreensis	13.76
Corynebacterium tuberculostearicum	7.96
Corynebacterium jeikeium	4.60
Corynebacterium massiliense	2.07
Brevibacterium paucivorans	1.19
Peptoniphilus olsenii	0.92
Finegoldia magna	0.85
Propioniferax innocua	0.50
Peptoniphilus ivorii	0.44
Dermabacter vaginalis	0.44
Corynebacterium frankenforstense	0.40
Anaerococcus octavius	0.24
Facklamia languida	0.07
Ignavigranum ruoffiae	0.04
Corynebacterium bovis	0.03
Helcococcus kunzii	0.03
Other	0.03
Moraxella osloensis	0.02

Anaerococcus provencensis	0.02
Alkalibacterium putridalgicola	0.01
Ercella succinigenes	0.01
Anaerococcus senegalensis	0.01
Corynebacterium falsenii	0.01
Streptococcus periodonticum	0.01
Anaerococcus nagyae	0.01
Miscellaneous unidentified	0.02
Total	100.00

Nail 10

**Nail 10 16S**	**Hits %**
Staphylococcus epidermidis	77.79
Corynebacterium massiliense	12.37
Corynebacterium bovis	2.72
Corynebacterium tuberculostearicum	1.56
Anaerococcus octavius	1.46
Anaerococcus nagyae	1.08
Aerococcus sanguinicola	1.07
Rothia koreensis	1.05
Anaerococcus urinomassiliensis	0.11
Finegoldia magna	0.08
Moraxella osloensis	0.06
Rubrobacter xylanophilus	0.06
Corynebacterium amycolatum	0.05
Peptoniphilus olsenii	0.04
Streptococcus thermophilus	0.04
Peptoniphilus grossensis	0.04
Deinococcus fonticola	0.03
Blautia wexlerae	0.03
Methylorubrum populi	0.02
Akkermansia muciniphila	0.02
Brevibacterium paucivorans	0.02
Staphylococcus argensis	0.02
Monoglobus pectinilyticus	0.02
Lachnospira eligens	0.02
Gemmiger formicilis	0.02
Peptoniphilus ivorii	0.02
Escherichia fergusonii	0.02
Phocaeicola dorei	0.01

Streptococcus periodonticum	0.01
Corynebacterium frankenforstense	0.01
Corynebacterium ihumii	0.01
Petrocella atlantisensis	0.01
Brevibacterium sediminis	0.01
Cutibacterium acnes	0.01
Bacteroides uniformis	0.01
Coprococcus comes	0.01
[*Clostridium*] *scindens*	0.01
Holdemanella biformis	0.01
Dialister invisus	0.01
Methylobacterium bullatum	0.01
Altererythrobacter dongtanensis	0.01
Neisseria mucosa	0.01
Corynebacterium glyciniphilum	0.01
Pseudoglutamicibacter cumminsii	0.01
Enterococcus hirae	0.01
Peptoniphilus lacrimalis	0.01
Miscellaneous unidentified	0.03
Total	100.00

Nail 11

**Nail 11. (Control 1) 16S**	**Hits %**
Staphylococcus epidermidis	80.34
Rothia koreensis	10.78
Corynebacterium tuberculostearicum	8.86
Moraxella osloensis	0.01
Miscellaneous unidentified	0.02
Total	100.00

Nail 12

**Nail 12 16S**	**Hits %**
Staphylococcus epidermidis	55.96
Rothia koreensis	30.94
Brevibacterium sediminis	4.65
Corynebacterium tuberculostearicum	4.30
Corynebacterium massiliense	1.92
Corynebacterium jeikeium	0.68
Dermabacter vaginalis	0.68
Peptoniphilus olsenii	0.28

Corynebacterium resistens	0.21
Anaerococcus octavius	0.10
Moraxella osloensis	0.07
Dietzia timorensis	0.06
Clavibacter michiganensis	0.02
Nesterenkonia cremea	0.02
Brevibacterium paucivorans	0.02
Finegoldia magna	0.02
Cutibacterium acnes	0.01
Kocuria uropygioeca	0.01
Other	0.01
Micrococcus luteus	0.01
Rubrobacter xylanophilus	0.01
Miscellaneous unidentified	0.03
Total	100.00

Nail 13

**Nail 13 16S**	**Hits %**
Staphylococcus epidermidis	48.84
Rothia koreensis	15.06
Corynebacterium tuberculostearicum	7.57
Corynebacterium jeikeium	5.65
Sphingobacterium daejeonense	5.27
Brevibacterium paucivorans	3.57
Dermabacter vaginalis	3.51
Clavibacter michiganensis	3.35
Brevibacterium sediminis	3.28
Ignavigranum ruoffiae	0.93
Corynebacterium glyciniphilum	0.75
Corynebacterium resistens	0.53
Dietzia timorensis	0.32
Anaerococcus octavius	0.32
Corynebacterium bovis	0.22
Anaerococcus urinomassiliensis	0.19
Micrococcus luteus	0.17
Propioniferax innocua	0.17
Sphingobacterium hotanense	0.10
Corynebacterium massiliense	0.04
Moraxella osloensis	0.03
Dermacoccus profundi	0.02
Staphylococcus lentus	0.02

Corynebacterium aurimucosum	0.01
Gordonia hongkongensis	0.01
Brachybacterium faecium	0.01
Gulosibacter chungangensis	0.01
Staphylococcus pasteuri	0.01
Finegoldia magna	0.01
Brevundimonas albigilva	0.01
Miscellaneous unidentified	0.02
Total	100.00

Nail 14

Nail 14 16S	Hits %
Staphylococcus epidermidis	99.14
Corynebacterium ihumii	0.56
Corynebacterium tuberculostearicum	0.07
Corynebacterium riegelii	0.05
Micrococcus luteus	0.05
Corynebacterium massiliense	0.02
Moraxella osloensis	0.01
Rubrobacter xylanophilus	0.01
Cutibacterium acnes	0.01
Ezakiella coagulans	0.01
Mycolicibacterium obuense	0.01
Porphyromonas pasteri	0.01
Miscellaneous unidentified	0.04
Total	100.00

Nail 15

Nail 15 16S	Hits %
Staphylococcus epidermidis	80.41
Rothia koreensis	17.85
Corynebacterium bovis	0.90
Corynebacterium jeikeium	0.26
Dermabacter vaginalis	0.21
Dermacoccus profundi	0.18
Corynebacterium tuberculostearicum	0.06
Cutibacterium acnes	0.02
Finegoldia magna	0.02
Moraxella osloensis	0.02
Rubrobacter xylanophilus	0.01

Corynebacterium amycolatum	0.01
Corynebacterium ihumii	0.01
Methylobacterium bullatum	0.01
Miscellaneous unidentified	0.02
Total	100.00

Nail 16

Nail 16 16S	Hits %
Staphylococcus epidermidis	56.95
Rothia koreensis	42.11
Dermabacter vaginalis	0.47
Staphylococcus lentus	0.23
Brevibacterium sediminis	0.15
Dietzia timorensis	0.03
Brevibacterium paucivorans	0.02
Corynebacterium jeikeium	0.01
Clavibacter michiganensis	0.01
Staphylococcus pasteuri	0.01
Miscellaneous unidentified	0.01
Total	100.00

Nail 17

Nail 17 16S	Hits %
Staphylococcus epidermidis	54.13
Rothia koreensis	23.33
Corynebacterium tuberculostearicum	6.28
Dermabacter vaginalis	3.52
Brevibacterium sediminis	3.51
Anaerococcus octavius	3.17
Corynebacterium massiliense	2.21
Corynebacterium resistens	1.06
Dermacoccus profundi	1.02
Peptoniphilus olsenii	0.53
Brevibacterium paucivorans	0.32
Facklamia languida	0.32
Dietzia timorensis	0.15
Staphylococcus lentus	0.13
Corynebacterium frankenforstense	0.10
Moraxella osloensis	0.07
Clavibacter michiganensis	0.03

Corynebacterium bovis	0.02
Brachybacterium faecium	0.02
Helcobacillus massiliensis	0.01
Other	0.01
Brevundimonas albigilva	0.01
Micrococcus luteus	0.01
Rubrobacter xylanophilus	0.01
Bacillus velezensis	0.01
Anaerococcus prevotii	0.01
Miscellaneous unidentified	0.02
Total	100.00

Nail 18

**Nail 18 16S**	**Hits %**
Staphylococcus epidermidis	71.85
Rothia koreensis	16.73
Corynebacterium jeikeium	3.03
Brevibacterium paucivorans	2.00
Corynebacterium tuberculostearicum	1.35
Corynebacterium massiliense	1.32
Corynebacterium resistens	1.14
Corynebacterium bovis	1.05
Corynebacterium amycolatum	0.56
Pseudoglutamicibacter cumminsii	0.52
Diplorickettsia massiliensis	0.12
[Pseudomonas] geniculata	0.09
Staphylococcus argensis	0.04
Brevibacterium sediminis	0.03
Propioniferax innocua	0.03
Staphylococcus simulans	0.03
Acinetobacter tjernbergiae	0.02
Anaerococcus octavius	0.01
Moraxella osloensis	0.01
Ezakiella massiliensis	0.01
Pseudomonas soli	0.01
Corynebacterium frankenforstense	0.01
Corynebacterium freiburgense	0.01
Dermabacter vaginalis	0.01
Finegoldia magna	0.01
Peptoniphilus obesi	0.01
Miscellaneous unidentified	0.02

Total	100.00

Nail 19

Nail 19 16S	Hits %
Moraxella osloensis	34.58
Rothia koreensis	14.80
Brevibacterium sediminis	11.79
Deinococcus fonticola	7.11
Corynebacterium resistens	6.94
Staphylococcus epidermidis	5.45
Deinococcus proteolyticus	4.63
Dermabacter vaginalis	4.62
Deinococcus taklimakanensis	2.83
Corynebacterium bovis	1.41
Dietzia timorensis	1.36
Brevundimonas albigilva	1.22
Finegoldia magna	1.08
Brevibacterium paucivorans	0.66
Staphylococcus lentus	0.49
Clavibacter michiganensis	0.42
Corynebacterium jeikeium	0.11
Roseomonas gilardii	0.09
Lysobacter oculi	0.09
Anaerococcus octavius	0.08
Ignavigranum ruoffiae	0.05
Gordonia hongkongensis	0.04
Dermacoccus profundi	0.04
Facklamia languida	0.04
Corynebacterium tuberculostearicum	0.02
Corynebacterium amycolatum	0.01
Cutibacterium acnes	0.01
Propioniferax innocua	0.01
[*Pseudomonas*] *geniculata*	0.01
Miscellaneous unidentified	0.01
Total	100.00

Nail 20

Nail 20 (Control 2) 16S	Hits %
Staphylococcus epidermidis	95.20
Rothia koreensis	3.10

Corynebacterium massiliense	0.58
Corynebacterium resistens	0.45
Corynebacterium tuberculostearicum	0.14
Moraxella osloensis	0.11
Anaerococcus octavius	0.10
Corynebacterium bovis	0.06
Finegoldia magna	0.05
Brevibacterium paucivorans	0.05
Rubrobacter xylanophilus	0.03
Dermabacter vaginalis	0.02
Corynebacterium jeikeium	0.02
Peptoniphilus grossensis	0.01
Haematomicrobium sanguinis	0.01
Streptococcus periodonticum	0.01
Brevibacterium sediminis	0.01
Corynebacterium ihumii	0.01
Cutibacterium acnes	0.01
Streptococcus anginosus	0.01
Veillonella dispar	0.01
Peptoniphilus ivorii	0.01
Peptoniphilus olsenii	0.01
Brevundimonas albigilva	0.01
Miscellaneous unidentified	0.02
Total	100.00
