# Supplementary material for: Metagenomics of Toenail Onychomycosis in Three Victorian Regions of Australia
Source: J Fungi (Basel). 2022 Nov 14;8(11):1198. doi: 10.3390/jof8111198 (PMC9698484; doi:10.3390/jof8111198)
Supplement: Supplementary file 1 [file jof-08-01198-s001.zip › Supplementary Tables S1, S4, S5, S6. docx.pdf]

Table S1 Primers used for sequencing by locus

Region	Primers (5′ to 3′)
ITS2 rRNA	ITS3 Forward GCA TCGA TGAAGAACGCAGC<br>ITS4 Reverse TCCTCCGCTTATTGATATGC
16S rRNA	Bakt_341 Forward CCTACGGGNGGCWGCAG<br>Bakt_805 Reverse GACTACHVGGGTATCTAATCC

Table S4 Predominant organism percentage of hits, with most likely infecting agent (MLIA).

Nail Number	Dermatophyte or NDM	% Hits	MLIA(s)
1	*T. rubrum*	98.11	*T. rubrum*
2	*T. rubrum*	95.53	*T. rubrum*
3	*T. rubrum*	99.93	*T. rubrum*
4	*T. mentagrophytes*	99.13	*T. mentagrophytes*
5	*A. versicolor*	99.4	*Aspergillus versicolor*
6	*T. mentagrophytes*	70.39	*T. mentagrophytes*
7	*T. mentagrophytes*	99.45	*T. mentagrophytes*
8	*A. sclerotigenum*	86.27	*Acremonium sclerotigenum*
9	*T. mentagrophytes*	90.23	*T. mentagrophytes*
10	*F. oxysporum*<br>*T. mentagrophytes*	86.27<br>12.81	Mixed
11 (Control 1)	*F. oxysporum*	95.77	-
12	*T. mentagrophytes*	94.73	*T. mentagrophytes*
13	*T. rubrum*	94.82	*T. rubrum*
14	*T. rubrum*	98.62	*T. rubrum*
15	*T. rubrum*	97.77	*T. rubrum*
16	*T. mentagrophytes*	90.43	*T. mentagrophytes*
17	*T. mentagrophytes*	97.63	*T. mentagrophytes*
18	*T. rubrum*	83.8	*T. rubrum*
19	*F. acetilerea*<br>*T. mentagrophytes*	51.92<br>47.55	Mixed
20 (Control 2)	*M. slooffiae*	93.57	-

Table S5 Nails with both *T. rubrum* and *T. mentagrophytes* present and showing the dominance of one dermatophyte over the other

Nail Number	Dermatophyte	% Hits
1	*T. rubrum*<br>*T. mentagrophytes*	98.11<br>0.65
4	*T. rubrum*<br>*T. mentagrophytes*	0.01<br>99.13
7	*T. rubrum*<br>*T. mentagrophytes*	0.02<br>99.45
10	*T. rubrum*<br>*T. mentagrophytes*	0.01<br>12.81
11 (Control 1)	*T. rubrum*<br>*T. mentagrophytes*	0.08<br>0.06
12	*T. rubrum*<br>*T. mentagrophytes*	0.02<br>94.73

Table S6 Correlations between results of sequencing the 16S region in DNA from nails.

**Correlations**	Nail 1	Nail 2	Nail 3	Nail 4	Nail 5	Nail 6	Nail 7	Nail 8	Nail 9	Nail 10	Nail 11	Nail 12	Nail 13	Nail 14	Nail 15	Nail 16	Nail 17	Nail 18	Nail 19
Nail 2	**0.917**																		
Nail 3	**0.788**	**0.917**																	
Nail 4	**0.904**	**0.99**	**0.937**																
Nail 5	**0.796**	**0.923**	**1**	**0.943**															
Nail 6	**0.573**	**0.662**	**0.727**	**0.678**	**0.729**														
Nail 7	**0.823**	**0.943**	**0.996**	**0.964**	**0.997**	**0.723**													
Nail 8	**0.867**	**0.921**	**0.815**	**0.894**	**0.824**	**0.649**	**0.838**												
Nail 9	**0.851**	**0.953**	**0.97**	**0.976**	**0.975**	**0.723**	**0.984**	**0.894**											
Nail 10	**0.78**	**0.908**	**0.987**	**0.928**	**0.987**	**0.72**	**0.983**	**0.815**	**0.966**										
Nail 11	**0.831**	**0.945**	**0.985**	**0.967**	**0.989**	**0.733**	**0.993**	**0.878**	**0.994**	**0.975**									
Nail 12	**0.909**	**0.969**	**0.87**	**0.984**	**0.88**	**0.638**	**0.911**	**0.896**	**0.949**	**0.869**	**0.928**								
Nail 13	**0.895**	**0.956**	**0.929**	**0.971**	**0.935**	**0.696**	**0.951**	**0.922**	**0.982**	**0.921**	**0.967**	**0.96**							
Nail 14	**0.787**	**0.917**	**1**	**0.937**	**1**	**0.731**	**0.996**	**0.815**	**0.97**	**0.987**	**0.985**	**0.87**	**0.929**						
Nail 15	**0.861**	**0.964**	**0.976**	**0.987**	**0.98**	**0.708**	**0.992**	**0.857**	**0.99**	**0.966**	**0.99**	**0.954**	**0.968**	**0.976**					
Nail 16	**0.885**	**0.926**	**0.802**	**0.952**	**0.814**	**0.578**	**0.855**	**0.824**	**0.897**	**0.798**	**0.869**	**0.986**	**0.915**	**0.802**	**0.913**				
Nail 17	**0.911**	**0.971**	**0.908**	**0.988**	**0.917**	**0.674**	**0.941**	**0.911**	**0.972**	**0.908**	**0.958**	**0.991**	**0.976**	**0.908**	**0.972**	**0.963**			
Nail 18	**0.864**	**0.963**	**0.972**	**0.987**	**0.977**	**0.707**	**0.989**	**0.867**	**0.994**	**0.965**	**0.99**	**0.957**	**0.975**	**0.972**	**0.998**	**0.915**	**0.974**		
Nail 19	0.334	0.281	0.115	0.251	0.121	0.067	0.148	0.288	0.177	0.115	0.157	0.287	0.224	0.114	0.187	0.3	0.262	0.191	
Nail 20	**0.801**	**0.927**	**0.999**	**0.947**	**1**	**0.727**	**0.998**	**0.824**	**0.976**	**0.987**	**0.989**	**0.885**	**0.937**	**0.999**	**0.983**	**0.821**	**0.921**	**0.979**	0.127

Bold=significant correlation.
